# *SMARCA4* deficiency-associated heterochromatin induces intrinsic DNA replication stress and susceptibility to ATR inhibition in lung adenocarcinoma

**DOI:** 10.1093/narcan/zcaa005

**Published:** 2020-05-01

**Authors:** Kiminori Kurashima, Hideto Kashiwagi, Iwao Shimomura, Ayako Suzuki, Fumitaka Takeshita, Marianne Mazevet, Masahiko Harata, Takayuki Yamashita, Yusuke Yamamoto, Takashi Kohno, Bunsyo Shiotani

**Affiliations:** Division of Cellular Signaling, National Cancer Center Research Institute, Chuo-ku, Tokyo 104-0045, Japan; Division of Cellular Signaling, National Cancer Center Research Institute, Chuo-ku, Tokyo 104-0045, Japan; Division of Molecular and Cellular Medicine, National Cancer Center Research Institute, Chuo-ku, Tokyo 104-0045, Japan; Department of Respirology, Graduate School of Medicine, Chiba University, Chiba-shi, Chiba 260-8670, Japan; Department of Computational Biology and Medical Sciences, Graduate School of Frontier Sciences, The University of Tokyo, Kashiwa-shi, Chiba 277-8562, Japan; Department of Functional Analysis, National Cancer Center Research Institute, Chuo-ku, Tokyo 104-0045, Japan; Division of Cellular Signaling, National Cancer Center Research Institute, Chuo-ku, Tokyo 104-0045, Japan; Graduate School of Agricultural Science, Tohoku University, Sendai-shi, Miyagi 980-0845, Japan; Laboratory of Molecular Genetics, Institute for Molecular and Cellular Regulation, Gunma University, Maebashi-shi, Gunma 371-8512, Japan; Division of Molecular and Cellular Medicine, National Cancer Center Research Institute, Chuo-ku, Tokyo 104-0045, Japan; Division of Genome Biology, National Cancer Center Research Institute, Chuo-ku, Tokyo 104-0045, Japan; Division of Cellular Signaling, National Cancer Center Research Institute, Chuo-ku, Tokyo 104-0045, Japan

## Abstract

The SWI/SNF chromatin remodeling complex regulates transcription through the control of chromatin structure and is increasingly thought to play an important role in human cancer. Lung adenocarcinoma (LADC) patients frequently harbor mutations in SMARCA4, a core component of this multisubunit complex. Most of these mutations are loss-of-function mutations, which disrupt critical functions in the regulation of chromatin architecture and can cause DNA replication stress. This study reports that LADC cells deficient in SMARCA4 showed increased DNA replication stress and greater sensitivity to the ATR inhibitor (ATRi) *in vitro* and *in vivo*. Mechanistically, loss of SMARCA4 increased heterochromatin formation, resulting in stalled forks, a typical DNA replication stress. In the absence of SMARCA4, severe ATRi-induced single-stranded DNA, which caused replication catastrophe, was generated on nascent DNA near the reversed forks around heterochromatin in an Mre11-dependent manner. Thus, loss of SMARCA4 confers susceptibility to ATRi, both by increasing heterochromatin-associated replication stress and by allowing Mre11 to destabilize reversed forks. These two mechanisms synergistically increase susceptibility of SMARCA4-deficient LADC cells to ATRi. These results provide a preclinical basis for assessing SMARCA4 defects as a biomarker of ATRi efficacy.

## INTRODUCTION

SWI/SNF chromatin remodeling complexes regulate gene transcription, DNA replication and DNA repair by organizing the chromatin architecture via ATP hydrolysis (1). There are three defined members of this subfamily: the canonical BRG-/BRM-associated factor (BAF), polybromo-associated BAF complexes and a newly identified non-canonical BAF (2,3). Increasing evidence indicates that these complexes have broad roles in tumor suppression (4–7), as inactivating mutations have been identified at high frequency in a variety of cancers (8). We and others found that *SMARCA4* (SWI/SNF-related, matrix-associated, actin-dependent regulator of chromatin, subfamily A, member 4) mutations were present in 7–8% of lung adenocarcinoma (LADC) patients (9,10). Recent studies showed that SWI/SNF complexes functionally antagonize transcription-silencing polycomb repressive complexes (PRCs), which regulate chromatin structure via post-translational histone modification (11), and that mutationally inactive SMARCA4 has no effect on PRCs (4,6). This resulted in imbalances between differentiation and self-renewal in the epigenetic regulation of gene transcription, thereby promoting tumorigenesis.

Although recent work has identified drug-susceptible driver mutations in lung cancer, including *ALK* (anaplastic lymphoma kinase) translocations and *EGFR* (epidermal growth factor receptor) mutations, LADC patients harboring SWI/SNF complex mutations often lack these driver gene mutations (12,13), a discrepancy that underscores the importance of developing new treatment strategies. Because most SWI/SNF complex mutations in tumor cells are loss-of-function mutations that cannot be targeted directly by therapeutic agents, vulnerabilities resulting from these mutations may be targets of treatment. Genetic analyses have shown the lethality of synthetic combinations of *SMARCA2* and *SMARCA4* (13,14) and of *ARID1A* and *ARID1B* (15). Although these synthetic lethal relationships suggest potential therapeutic opportunities, approaches targeting residual SWI/SNF complexes in cancers carrying SWI/SNF mutations remain under development (16,17).

In addition to acting as transcriptional regulators, SWI/SNF complexes also play roles in DNA repair. SWI/SNF complexes are recruited to damaged chromatin via a BRIT1-mediated mechanism (18) or via direct interactions between SMARCA4, γH2AX nucleosomes and acetylated histone H3 (19). These recruited complexes are functionally important for both non-homologous end joining and homologous recombination (HR) (20). Moreover, some SWI/SNF complex subunits, including ARID1A and ARID1B, were recently reported to be required for non-homologous end joining (21), whereas SMARCA4 was found to promote HR by facilitating the replacement of RPA with RAD51 (22), suggesting that SWI/SNF complexes protect genome integrity. SWI/SNF complexes are also involved in chromatin binding of topoisomerase IIα (23). Deletion of *SMARCA4* in mouse embryonic stem cells led to reductions in replication fork progression rates, anaphase bridge formation, G2/M arrest, micronuclei formation and aneuploidy (23–25), phenotypes characteristic of the mitotic entry of cells with incompletely replicated genomes under conditions of replication stress (26,27). Defects in critical functions of SWI/SNF complexes during the DNA damage response (DDR) and in the regulation of chromatin architecture likely result in DNA replication stress, leading to genomic instability and tumor development.

DNA replication stress is a driving force in the generation of genome instability during early stages of cancer development (28), as well as being a marker of developed cancer (29). To maintain genomic stability, cells have developed sophisticated signaling pathways to resolve DNA damage or DNA replication stress. One of the key mediators of responses to DNA replication stress is the ataxia telangiectasia-mutated and Rad3-related (ATR) kinase, which induces cell cycle arrest and facilitates DNA repair via its downstream targets (30–32). Tumor cells in many types of cancer are highly dependent on ATR signaling for survival, making ATR a promising target for cancer therapy. Tumor cells with compromised DNA repair pathways or DNA damage checkpoints rely on HR, and cells with increased DNA replication stress are particularly sensitive to ATR inhibition (33,34). Depletion of functional ATR sensitizes cancer cells to oncogene-induced replication stress, inhibiting tumor growth and inducing cell death (35–37). Importantly, hypomorphic ATR signaling defects were sufficient to induce synthetic lethality in oncogenic RAS-driven tumors, while having minimal effects on bone marrow and intestinal homeostasis (37). These findings suggest that low ATR activity might be sufficient to sustain the viability of highly proliferative adult tissues, as well as suggesting that partial inhibition of ATR kinase activity may be sufficient to induce robust and selective toxicity in cancer cells subjected to elevated DNA replication stress.

The present study reports that loss of SMARCA4, a catalytic subunit of SWI/SNF complexes, sensitizes LADC cells to an ATR inhibitor (ATRi). The synthetic lethal interaction between *ATR* and *SMARCA4* was observed both *in vitro* and *in vivo*. Mechanistically, ATR inhibition in SMARCA4-deficient cells with increased heterochromatin formation was found to increase exposure of single-stranded DNA (ssDNA), which is initiated by Mre11-dependent resection of nascent DNA at reversed forks, resulting in replication catastrophe (RC). These findings suggest that ATR plays crucial roles in stabilizing stalled forks and in maintaining cell viability in SMARCA4-deficient LADC cells. These findings also provide a scientific basis for targeting *SMARCA4* mutations in LADC via pharmacological inhibition of ATR activity.

## MATERIALS AND METHODS

### Cell lines

The LADC cell lines (A427, A549, H1299, H1650, H1819, H1975, H2126, H2228, H2347, H322, RERF-LC-MS, RERF-LC-OK, PC9 and PC14) are described in Supplementary Table S1. All cells were maintained in RPMI 1640 medium, except for A549 cells, which were maintained in Dulbecco’s modified Eagle’s medium (DMEM), with both media supplemented with 10% heat-inactivated fetal bovine serum (FBS; HyClone, GE Healthcare), 100 U/ml penicillin and 100 μg/ml streptomycin (Nacalai Tesk) at 37°C under 5% CO_2_. A427, A549 and H1299 cells stably expressing SMARCA4 were generated by infection of these cells with SMARCA4-expressing lentivirus, followed by selection with 10 μg/ml blasticidin (Wako). Immortalized small airway epithelial cells (SAECs) derived from normal SAECs were kindly provided by Dr Kiyono, and maintained in BronchiaLife Basal Medium supplemented with the compounds in the LifeFactors kit (Lifeline Cell Technology).

### Lentivirus preparation

HEK293T cells were cultured in DMEM containing 10% FBS at 37°C under 5% CO_2_. Cells were seeded at a density of 2 × 10^5^ ml^−1^ 24 h before transfection, and HEK293T co-transfected 1:1:1 with the plasmids pCAG-HIVgp, pCMV-VSV-G-RSV-Rev and either CSII-CMV-MCS-IRES2-Bsd/SMARCA4 or CMII-CMV-MCS-IRES2-Bsd, using polyethylenimine to produce lentivirus. Forty-eight hours later, medium containing each lentivirus was collected and concentrated with a Lenti-X concentrator (Clontech) in accordance with the manufacturer’s instructions.

### Plasmid construction

cDNA encoding full-length human SMARCA4 was amplified via polymerase chain reaction (PCR) using pCMV-HA-BRG1 as a template, and the primers 5′-GTAGCTAGCCACCATGTCCACTCCAGACCCACCCCTGGG-3′ (forward, with an NheI site) and 5′-GATACCGGTTCAGTCTTCTTCGCTGCCACTTCCTGAGCG-3′ (reverse, with an AgeI site). The PCR product was inserted into NheI/AgeI-digested CMII-CMV-MCS-IRES2-Bsd plasmid (RIKEN) to generate CSII-CMV-MCS-IRES2-Bsd/SMARCA4.

### RNA interference

The siRNAs siSMARCA4 #1 and #2 (s13139 and s13141), siSMARCAL1 #1 and #2 (s531775 and s531776), siCtIP #1 and #2 (s11849 and s11851), siHP1β #1, #2 and #3 (s21549, s21550 and s21551), siSMARCA2 #1 and #3 (s13133 and s13135), sip53 #1 and #3 (s605 and s607), and siSLX4 #1, #2 and #3 (s39052, s39053 and s39054) were purchased from Thermo Fisher Scientific (Silencer Select). Cells were transfected with siRNAs by reverse transfection with Lipofectamine RNAiMax (Invitrogen) and treated with various drugs 48 h later.

### Chemicals

The ATRi VE822 and the EZH2 inhibitor GSK-126 were purchased from Selleck, and the ATM inhibitor KU55933 and the DNA-PK inhibitor NU7441 from Merck Millipore. The Mre11 inhibitor Mirin, 5-bromo-2′-deoxyuridine (BrdU), 5-chloro-2′-deoxyuridine (CldU), 5-iodo-2′-deoxyuridine (IdU) and chloroquine (CQ) were purchased from Sigma-Aldrich. Cisplatin was purchased from Wako, and camptothecin (CPT) was purchased from Abcam.

### Western blot analysis

Cells were lysed in sodium dodecyl sulfate (SDS) buffer (0.125 M Tris–HCl, pH 6.8, 4% SDS, 10% sucrose, 0.01% bromophenol blue and 0.2 M dithiothreitol (DTT)), and the lysates were incubated at 95°C for 5 min. The protein concentrations in the samples were estimated by the Bradford assay (XL-Bradford, Apro Science). Equal amounts of protein were resolved by SDS-PAGE and transferred to polyvinylidene difluoride (PVDF) membranes (Millipore) at 150 mA for 16 h at 4°C. The membranes were blocked by incubation with 5% skim milk in TBST (1× Tris-buffered saline supplemented with 0.1% Tween 20), and incubated with primary antibodies in 5% skim milk in TBST. The primary antibodies included anti-α-tubulin (1/10000, MBL, PM054), anti-APOBEC3B (1/1000, Abcam, ab184990), anti-ARID1A (1/1000, GeneTex, GTX129433), anti-ATM (1/1000, Cell Signaling Technology, #2873), anti-ATR (1/1000, GeneTex, GTX128146), anti-BRCA1 (1/200, Santa Cruz Biotechnology, sc-6954), anti-BRCA2 (1/500, Merck, OP-95), anti-CtIP (1/1000, Cell Signaling Technology, #9201), anti-ERCC1 (1/1000, GeneTex, GTX129282), anti-HP1β (1/1000, Cell Signaling Technology, #8676), anti-p21 (1/500, BD Pharmingen, 55428), anti-p53 (1/1000, Santa Cruz Biotechnology, sc-126), anti-Rad17 (1/1000, GeneTex, GTX100107), anti-phospho-Rad17 (S645) (1/1000, Bethyl Laboratories, A300-153A), anti-Rad51 (1/1000, Abcam, ab133534), anti-SLX4 (1/1000, Bethyl Laboratories, A302-270A), anti-SMARCA2 (1/1000, Cell Signaling Technology, #11966), anti-SMARCA4 (1/1000, GeneTex, GTX80217) and anti-SMARCAL1 (1/1000, Cell Signaling Technology, #44717). After three washes with TBST, the membranes were incubated with horseradish peroxidase-conjugated secondary antibodies in 5% skim milk in TBST for 1 h at room temperature (RT). After four washes in TBST, the blots were developed using the Western Lightning Plus ECL reagent (PerkinElmer) according to the manufacturer’s instructions and imaged using a LAS 3000 luminescent image analyzer (FujiFilm).

### Cell viability assays

Cells were seeded in 96-well plates. Twenty-four hours later, the cells were treated with VE822 and/or cisplatin and cultured for 5–7 days unless otherwise indicated. Cell viability was assessed using the PrestoBlue cell viability reagent (Invitrogen). After removing the culture medium, 100 μl of diluted PrestoBlue reagent was added to each well, followed by incubation for 30 min at 37°C under 5% CO_2_. Fluorescence, with excitation at 560 nm and emission at 600 nm, was measured using PrestoBlue reagent and a microplate reader (Synergy H1 or H4, Biotek). The area under the curve (AUC) was calculated using Prism 7 software (GraphPad).

### Estimation of G1 phase duration

G1 phase duration was estimated as the doubling time multiplied by the G1 phase ratio. The doubling time was calculated using the following formula: log 2/{log(*N*_*b*_/*N*_*a*_)/*T*_*b*−*a*_}, where *N*_*a*_ and *N*_*b*_ are the numbers of cells at times *a* and *b*, respectively, and *T*_*b*−*a*_ is the time between measurements. The percentages of cells in G1 phase were calculated based on flow cytometry analysis.

### Flow cytometry

Logarithmically growing cells were fixed overnight in 70% ethanol at −30°C. After two washes in phosphate-buffered saline (PBS), the cells were stained with 50 μg/ml propidium iodide (PI) in the presence of 100 μg/ml RNase A for 30 min at RT. Data were acquired and analyzed on a SONY Cell Analyzer EC800. The fraction of cells in G1 phase was determined by PI staining.

### Immunofluorescence assays

Cells were pre-extracted with 0.05% or 0.25% Triton X-100 in PBS for 5 min on ice, fixed in 4% paraformaldehyde in PBS for 10 min at RT, washed with PBS, post-extracted in 0.25% Triton X-100 in PBS for 10 min at RT and blocked with 3% bovine serum albumin (BSA) in PBS. The cells were subsequently incubated at 37°C for 1 h with anti-γH2AX (Cell Signaling Technology, #9718), anti-RPA2 (Abcam, ab2175), anti-Mre11 (GeneTex, GTX70212), anti-trimethyl-histone H3 (Lys9) (Merck, 07442) or anti-HP1β (Cell Signaling Technology, #8676) diluted in PBS containing 3% BSA and 0.05% Tween 20. The cells were subsequently washed three times and incubated with secondary antibodies conjugated to the appropriate fluorophore (Alexa-488 or Alexa-594) for 1 h at RT. After three additional washes, the cells were stained with 4′,6-diamidino-2-phenylindole (DAPI) and mounted with Vectashield (Vector Laboratories). Proximity ligase assays (PLAs) were performed with the indicated primary antibodies diluted 1:500 (SMARCAL1 and HP1α; Merck, 05-689) and Duolink In Situ PLA kits (Sigma-Aldrich). Immunofluorescence images were obtained with a fluorescence microscope (BZ-9000, Keyence) or a confocal laser scanning microscope (TCS SP8, Leica).

### Detection of ssDNA in S-phase cells

To visualize ssDNA, cells were cultured in 10 μM BrdU for 48 h and pulse-labeled with 10 μM 5-ethynyl-2′-deoxyuridine (EdU) for 30 min to detect S-phase cells. After three washes with PBS, the cells were treated with ATRi for 2 h, pre-extracted in 0.05% Triton X-100 in PBS, fixed and post-extracted as described for the immunofluorescence assays. The cells were processed with the Click-iT Plus EdU Alexa Fluor 594 Imaging Kit according to the manufacturer’s instructions (Thermo Fisher Scientific). The cells were subsequently incubated at 37°C for 1 h with an anti-BrdU antibody (1/500, GE Healthcare, RPN20AB) that does not recognize EdU, washed three times and incubated with Alexa-488-conjugated secondary antibody. The cells were washed an additional three times, stained with DAPI and mounted with Vectashield. Immunofluorescence images were obtained with a fluorescence microscope (BZ-9000).

### Quantitative fluorescence analysis of individual cells

Quantitative fluorescence analyses were performed as previously described (38) with modifications. The BrdU, EdU, γH2AX, RPA2, Mre11, HP1β, H3K9me3 and DAPI signals in individual cells were quantified using the BZ-X analyzer. To quantify the ssDNA levels in S-phase cells, the BrdU signals of 200 randomly selected EdU-positive DAPI-stained nuclei were measured. To quantify the induction of ssDNA by ATRi in the SAEC and LADC cell lines, a threshold was set for each cell line above 97.5% of dimethyl sulfoxide (DMSO)-treated control cells. The ATRi-treated cells that showed ssDNA signals above these thresholds were scored as positive.

### DNA fiber assay

The DNA fiber assays were performed as described (39,40). Cells were sequentially labeled with 100 μM IdU for 10 min and 100 μM CldU for 20 min. The labeled cells were trypsinized and mixed with a 5–10-fold excess of unlabeled cells. After one wash with medium, the cells were fixed in fixative solution (3:1 methanol/acetic acid), resuspended in this solution and spotted onto slides. After drying, the slides were immersed in lysis solution (200 mM Tris–HCl, pH 7.5, 50 mM ethylenediaminetetraacetic acid (EDTA) and 0.5% SDS) for 15 min at 37°C. The DNA fibers released from the cells were extended by tilting the slides in a high-humidity chamber over 30 min. The slides were immersed in fixative solution for 2 min and washed in distilled water. To denature the DNA fibers, the slides were immersed in 2.5 M HCl for 80 min and washed three times in PBS. After blocking with PBS containing 5% BSA for 20 min, the slides were incubated for 2 h at RT with anti-IdU (1/100, BD Biosciences, 347580) and anti-CldU (1/25, Abcam, ab6326) antibodies in PBS containing 5% BSA to label nascent DNA, followed by three washes with PBS and incubation at RT for 1 h with anti-rat IgG conjugated with Alexa Fluor 488 and anti-mouse IgG conjugated with Alexa Fluor 555 in PBS containing 5% BSA. The slides were washed three times in PBS and mounted with Vectashield. Images were captured with a fluorescence microscope (BZ-9000, Keyence) and analyzed using ImageJ software (National Institutes of Health). Micrometers were converted to kilobase pairs by multiplying the number of micrometers by 3.5 kb. Fork velocity was measured as the length of continuous red (IdU) and green (CldU) tracks for forks that moved throughout the pulse labeling divided by the labeling time (30 min). To measure fork velocity, at least 200 forks were examined in each sample. Sister fork asymmetry was measured as the ratio of the lengths of the left- and right-moving sister forks (green tracks in green–red–green tracks). Asymmetric forks were defined as sister forks differing >30% in length. To score asymmetric forks, at least 50 forks were examined in each sample.

### Animal studies

All mouse experiments were approved by the National Cancer Center Research Institute of Laboratory Animal Research (No. T18-009). A549 and H1975 cells were injected into the right flanks of 5-week-old female BALB/c nude mice (Charles River Laboratories Japan), together with matrigel/PBS (1.0 × 10^6^ cells, 50% final concentration). Five days later, the mice were randomly separated into two groups (*n* = 8 for A549 control and *n* = 7 for others) and treated with VE822 (60 mg/kg) or vehicle alone (10% d-α-tocopherol polyethylene glycol 1000 succinate) by oral gavage. Tumor sizes were measured with a Vernier caliper. Tumors were harvested 22 days after inoculation of cancer cells and their weight was measured.

### Immunohistochemistry

Xenograft tumors were fixed in 4% paraformaldehyde, embedded in paraffin and sectioned. The sections were dewaxed in xylene and rehydrated through a graded ethanol series (100%, 80% and 70%). Antigen was retrieved by boiling the specimens at 98°C for 45 min in 1/200 diluted ImmunoSaver (Nissin EM), and endogenous peroxidase activity was blocked by incubation with 3% H_2_O_2_ for 15 min. The sections were permeabilized with 0.1% Triton X-100 in PBS for 15 min and blocked with blocking solution (DAKO, Protein Block Serum Free) for 30 min. The sections were incubated with anti-γH2AX (Cell Signaling Technology, #9718) or anti-Ki-67 (Abcam, ab16667) diluted in DAKO Real Diluent at 4°C for 16 h, washed three times and stained using ImmPRESS IgG-peroxidase kits (Vector Laboratories) and metal-enhanced DAB substrate kits (Thermo Fisher Scientific). After counterstaining with hematoxylin, the sections were dehydrated and mounted. Images were obtained using a BZ-X700 microscope (Keyence) and analyzed by ImageJ software.

### Statistical analyses

Results were compared by one-way analysis of variance (ANOVA) or the Mann–Whitney *U*-test. All statistical analyses were performed using Prism 7 software, with *P* < 0.05 considered statistically significant. Statistical significance was denoted as follows: *P < 0.05, **P < 0.01, ***P < 0.001, ****P < 0.0001.

## RESULTS

### SMARCA4-deficient LADC cells are selectively sensitized to ATR inhibition

**Figure 1.**
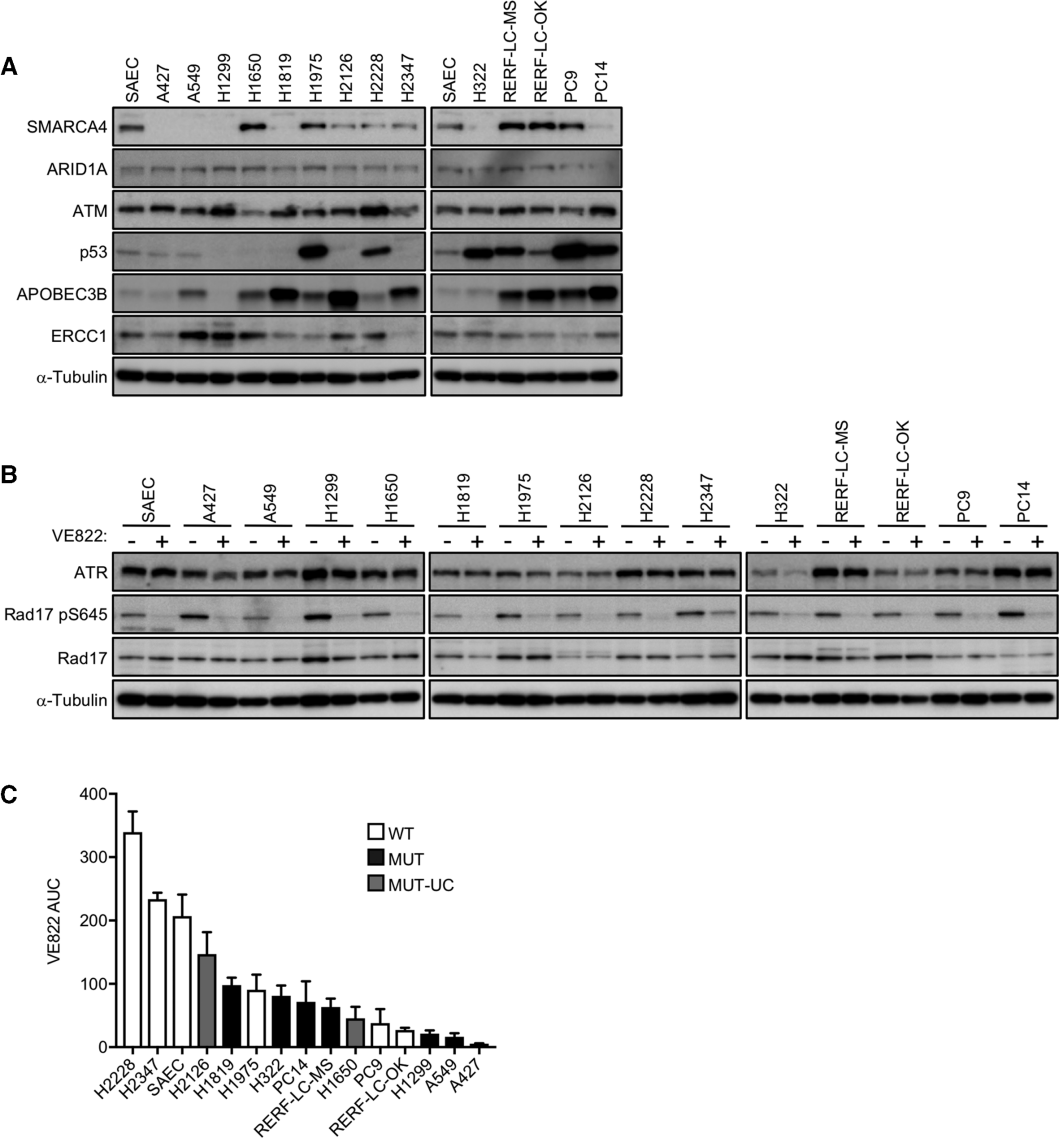
SMARCA4-deficient cells are highly sensitive to ATRi. (**A**) LADC cells and SAECs were lysed and subjected to western blot analysis with the indicated antibodies. α-Tubulin was used as a loading control. (**B**) LADC cells and SAECs were treated with DMSO or 1 μM ATRi for 2 h, and the levels of ATR and pRad17 were analyzed by western blotting. (**C**) LADC cells and SAECs expressing SMARCA4^WT^ (white), SMARCA4^MUT^ (black), or SMARCA4^MUT-UC^ (gray) were treated with ATRi (0.04–5 μM) for 6 days, and cell viability was measured by the PrestoBlue assay. The AUCs of each cell line are arranged in order. WT, wild type; MUT, deleterious mutation; MUT-UC, uncharacterized mutation. The values represent the mean ± standard deviation (SD) of three independent experiments.

A recent study testing the effects of inhibition of ATR kinase, a master regulator of the DDR, found that ATR was an attractive target for cancer therapy, as many cancers have defects in certain components of the DDR that render them highly dependent on the remaining DDR pathways for survival (41). To investigate whether SMARCA4 defects result in greater susceptibility to ATRi, we tested the effects of ATRi VE822, a close analog of the widely used ATRi VE821, on 14 LADC cell lines for which multi-omics data are available (DBKERO; http://kero.hgc.jp/), as well as on normal lung SAECs (42). These 14 LADC cell lines included five expressing wild-type SMARCA4 (SMARCA4^WT^; H1975, H2228, H2347, RERF-LC-OK and PC9), seven expressing mutated SMARCA4 (SMARCA4^MUT^ cells, no SMARCA4: A427, A549, H1299, H1819 and H322; low SMARCA4 expression: PC14; and helicase ATP-binding domain largely deleted-SMARCA4 expression: RERF-LC-MS) and two expressing as yet uncharacterized SMARCA4 mutations (SMARCA4^MUT-UC^ cells; H1650 and H2126) (Figure 1A; Supplementary Tables S1 and S2; Supplementary Figure S1A). Testing with ATRi VE822 showed excellent ATR selectivity, as indicated by specific inhibition of Rad17 phosphorylation at Ser 645 (p-Rad17) (43) (Supplementary Figure S1B). In contrast to the effects of the ATRi, inhibitors of two other DDR kinases, ATM (KU55933) and DNA-PK (NU7441), had no effect on p-Rad17 accumulation. All of these cell lines expressed basal levels of p-Rad17 (Figure 1B), suggesting modest activation of ATR signaling due to intrinsic replication stress experienced during normal proliferation, independent of any extrinsic stress. Regardless of the levels of ATR expression, the decreased p-Rad17 levels in the presence of ATRi provided evidence of effective ATRi uptake by all of the tested cell lines (Figure 1B).

We also investigated whether SMARCA4^MUT^ cells were more dependent on ATR for growth than SMARCA4^WT^ cells by testing the viability of the 14 LADC cell lines and the control SAEC line. We found that the three most responsive cell lines were SMARCA4^MUT^ cells (A427, A549 and H1299), whereas most of the SMARCA4^WT^ cells were largely resistant to ATRi (Figure 1C). Similar trends were not observed when cisplatin sensitivity was tested (Supplementary Figure S1C). The ATRi sensitivity of several SMARCA4^WT^ and SMARCA4^MUT-UC^ cells (PC9 and H1650) was similar to that of SMARCA4^MUT^ cells. These cells harbor EGFR-activating mutations as drivers, which correlate with a Fanconi anemia (FA)-like cellular phenotype reported to be a synthetic lethal factor for ATR inhibition (44,45). ATRi sensitivity caused by the FA-like phenotype was independent of the response to replication stress as discussed later. Collectively, these results suggested that ATR suppresses increased intrinsic replication stress in proliferating SMARCA4^MUT^ cells, rendering them highly sensitive to ATRi.

### ATRi-induced replication catastrophe is enhanced by cisplatin-driven replication stress

**Figure 2.**
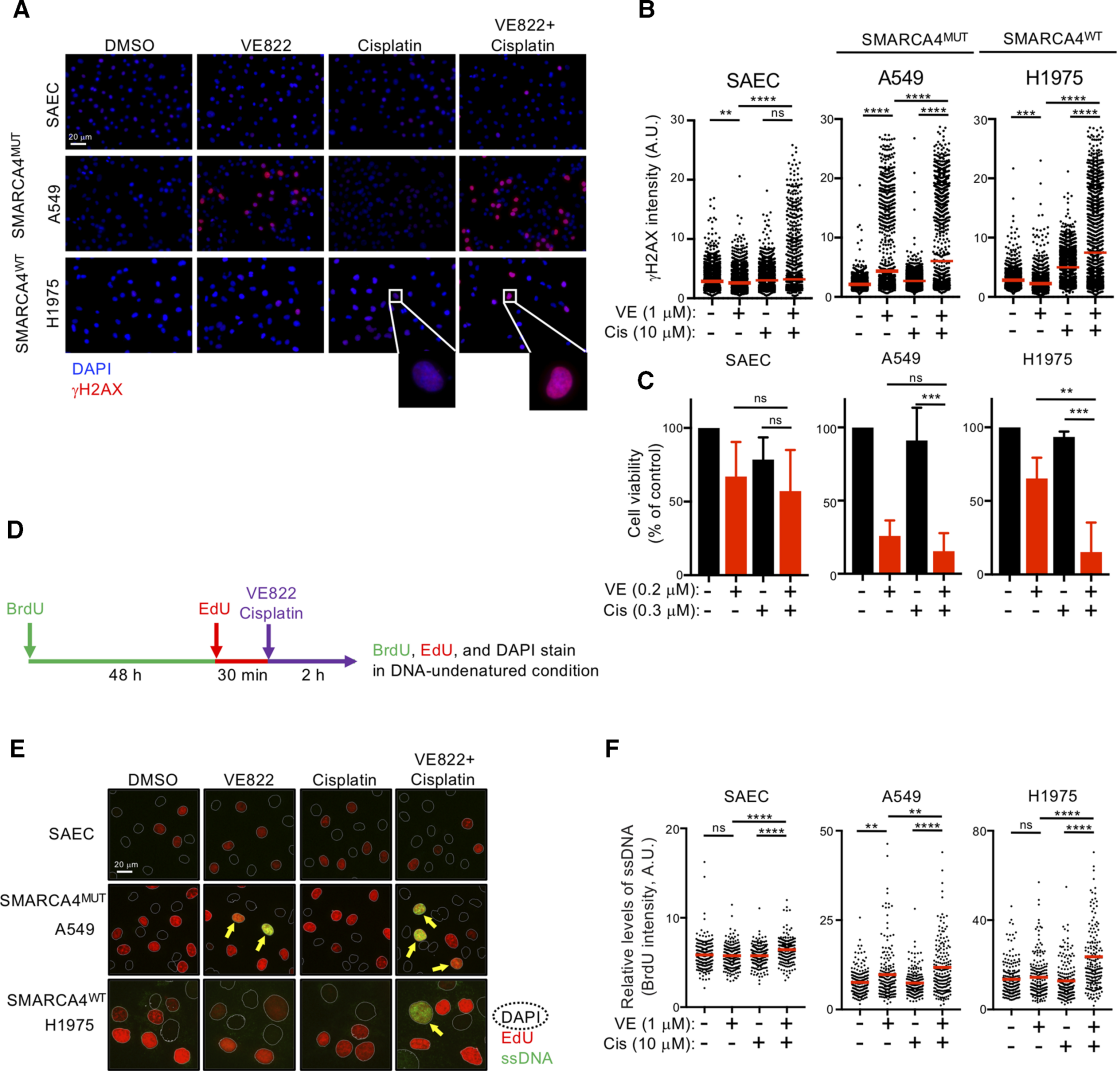
Cisplatin-mediated DNA replication stress synergistically enhances ATRi-induced RC. (**A**, **B**) Cells were treated with the indicated drugs for 8 h and immunostained with anti-γH2AX antibody, and their nuclei were counterstained with DAPI. (**A**) Representative image. Scale bar: 20 μm. (**B**) Quantification of the γH2AX intensities of 2000 cells treated with ATRi and cisplatin. Red bars represent mean intensities. Representative results of two independent reproducible experiments are shown. (**C**) Cells were treated with ATRi and/or cisplatin at the indicated concentrations for 6 days and cell viability was measured by the PrestoBlue assay. Each value represents the mean ± SD of three independent experiments. (**D**) Schematic representation of the experimental design used to analyze the exposed ssDNA by BrdU staining. Cells were incubated with 10 μM BrdU for 48 h, followed by 10 μM EdU for 30 min and ATRi and/or cisplatin for 2 h. The exposed ssDNA was immunostained with anti-BrdU (green) antibody under native conditions and the S-phase and total nuclei were counterstained with EdU (red) or DAPI (dashed line), respectively. (**E**) Cells were treated with ATRi and cisplatin as indicated, and the exposed ssDNA was analyzed. Scale bar: 20 μm. (**F**) Quantification of the ssDNA (BrdU) intensities of 200 S-phase cells (EdU positive) (**E**). Red bars represent the mean intensity. Representative results of two independent reproducible experiments are shown.

To understand the basis of ATRi toxicity in LADC cells, we analyzed the levels of DNA damage-induced γH2AX. Eight hours after ATRi treatment (1 μM), several types of SMARCA4^MUT^ cells (A549, A427 and H1299) became strongly pan-nuclear positive for γH2AX, an indicator of RC-associated DNA damage (46), whereas most of the SMARCA4^WT^ cells (H1975, H2228 and PC9) were only weakly positive (Figure 2A and B; Supplementary Figure S2A). By contrast, cisplatin treatment (10 μM) induced typical DNA damage-induced foci in both SMARCA4^WT^ and SMARCA4^MUT^ cells. Co-treatment of both SMARCA4^MUT^ and SMARCA4^WT^ cells with ATRi and cisplatin increased the number of pan-nuclear γH2AX-positive cells but not the number of cells positive for discernible foci (Figure 2A). These observations suggest that cisplatin-driven replication stress enhances RC-associated DNA damage induced by ATRi (Figure 2A and B). The RC-associated DNA damage phenotype induced by high doses was closely associated with cell viability observed when cells were treated with relatively low concentrations of drugs (ATRi, 0.1–0.4 μM; cisplatin, 0.3 μM) (Figure 2C and Supplementary Figure S2B). Treatment with ATRi alone for 6 days was sufficient to reduce the viability of SMARCA4^MUT^ but not SMARCA4^WT^ cells. Although treatment with 0.3 μM cisplatin alone did not robustly reduce the viability of most of these cell lines, except for PC9 cells, combination treatment with both agents synergistically killed the cells. Treatment of SAECs with either ATRi or cisplatin had no effect on the number of γH2AX-positive cells, whereas treatment with both induced a limited but significant increase in the number of pan-nuclear γH2AX-positive cells, indicating a modest sensitivity to ATRi/cisplatin combination treatment (Figure 2A–C).

The level of replication stress was estimated by measuring the abundance of exposed ssDNA. To visualize ssDNA in S-phase cells, their DNA was first labeled with BrdU for 48 h. The cells were subsequently released into EdU-containing media to label the S-phase cells for 30 min prior to drug treatment. Finally, native BrdU staining was analyzed (Figure 2D). ATRi treatment significantly increased the abundance of exposed ssDNA and chromatin-bound RPA in SMARCA4^MUT^ cells (A549) but not in SMARCA4^WT^ cells (Figure 2E and F; Supplementary Figure S2C). Interestingly, ATRi/cisplatin combination treatment greatly increased the abundance of exposed ssDNA in all of the tested cell lines, whereas treatment with cisplatin did not (Figure 2E and F). These results suggest that the effects of ATR inhibition synergize with those of DNA-damaging chemotherapeutic agents, such as cisplatin, to kill cancer cells, most likely via a cisplatin-dependent increase in replication stress rather than via elevated cisplatin-induced DNA damage in the absence of ATR activity. This hypothesis further suggests that SMARCA4^MUT^ cells experience higher intrinsic replication stress that leads to greater ATRi susceptibility.

### SMARCA4-deficient cells show elevated intrinsic replication stress strongly associated with ATRi sensitivity

**Figure 3.**
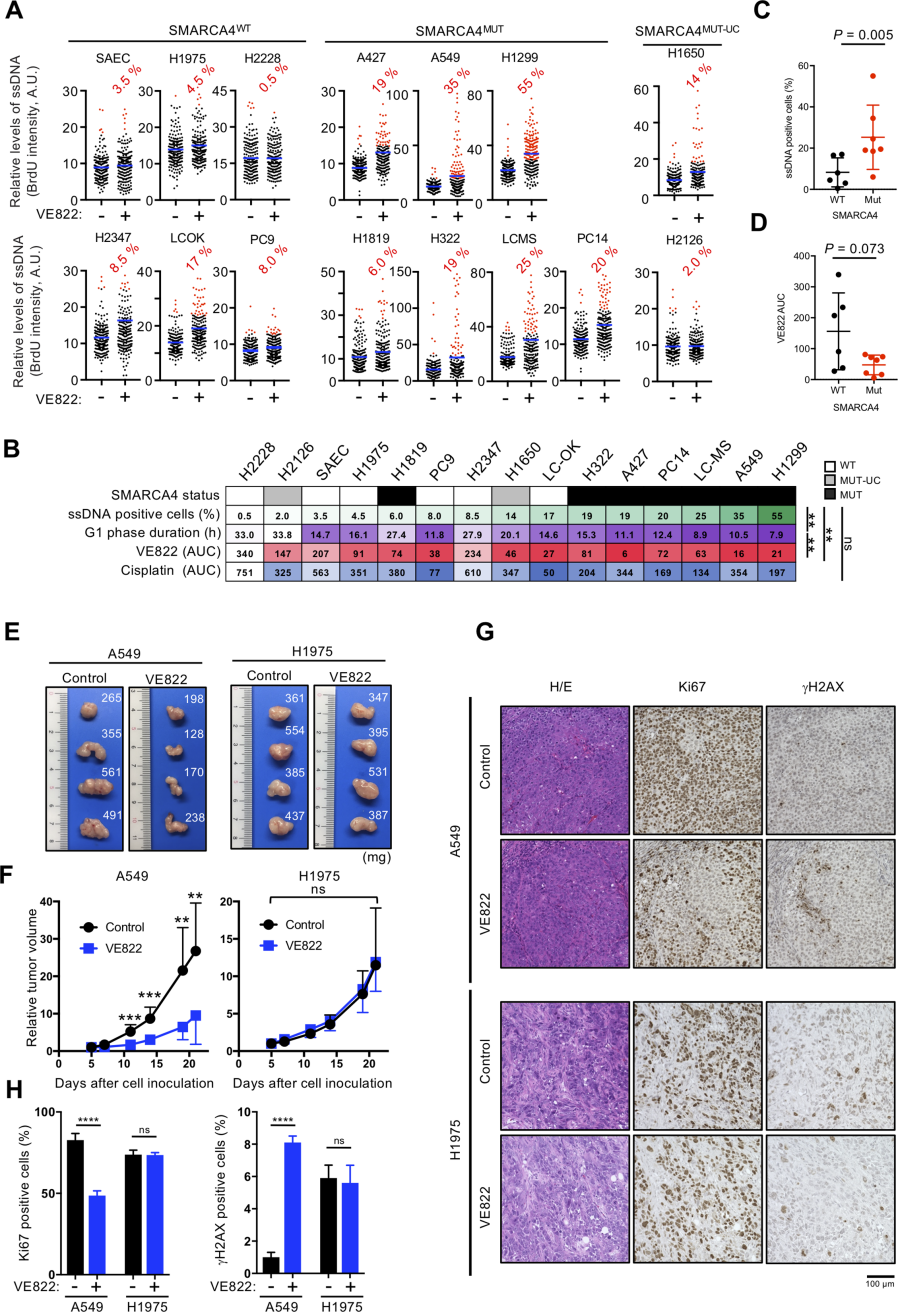
SMARCA4 status correlates with ATRi efficacy *in vitro* and *in vivo*. (**A**) Quantification of ssDNA intensities of 200 S-phase cells. Blue bars represent the mean intensity. ATRi induction of ssDNA was quantified by setting a threshold for each cell line above 97.5% of DMSO-treated control cells. The ATRi-treated cells that showed ssDNA signals above these thresholds were scored as positive (red dots), and the percentages of ssDNA-positive cells are shown. (**B**) Order of the percentage of ssDNA-positive cells and G1 phase duration in the assayed cell lines. The AUCs for ATRi and cisplatin are indicated. SMARCA4 status is shown above each. WT, wild type; MUT, deleterious mutation; MUT-UC, uncharacterized mutation. Correlation analyses were performed using Pearson’s coefficient. LADC cells and SAECs were divided into two groups (SMARCA4-WT or -MUT) and the percentages of ssDNA-positive cells (**C**) and AUCs for ATRi (**D**) were determined. MUT-UC cells were excluded. (**E**–**H**) A549 and H1975 cells were injected into the right flanks of BALB/c nude mice. Five days later, the mice were treated with ATRi or vehicle by oral gavage, as described in the legend to Supplementary Figure S3E. (**E**) Representative images of dissected tumors. (**F**) Quantitative analysis of tumor progression after 5 days. Data were normalized to pretreatment tumor mass and expressed as mean ± SD (*n* = 8 for A549 control, *n* = 7 for others). (**G**) Representative images of H&E, Ki-67 and γH2AX staining of tumor sections. Scale bar: 100 μm. (**H**) Quantification of Ki-67- and γH2AX-positive cells in tumor sections. The results represent the mean ± SD of three independent experiments.

Replication stress stimulates a variety of responses, most of which result in increased ssDNA accumulation. In unperturbed cells, intrinsic replication stress may induce a small but significant amount of ssDNA, which can modestly activate ATR to suppress excessive ssDNA accumulation (Figure 1B). To address the levels of intrinsic replication stress in LADC cells, ATRi-induced ssDNA was analyzed as described earlier. Treatment with ATRi for 2 h increased the number of ssDNA-positive S-phase A549 and H1299 (SMARCA4^MUT^) cells, but had no effect on the number of ssDNA-positive S-phase H1975 and H2228 (SMARCA4^WT^) cells (Supplementary Figure S3A). The percentages of ATRi-induced ssDNA-positive cells in a panel of 14 LADC cell lines and control SAECs were strongly associated with ATRi (*P* = 0.002) but not with cisplatin (n.s.) sensitivity (Figure 3A and B).

Shorter G1 phases lead to premature entry into S phase preceding the firing of intragenic origins, a mechanism of oncogene-induced DNA replication stress (47). To determine the duration of G1 phase in these cell lines, we tested their cell cycle states and doubling times (Supplementary Figure S3B–D), finding that shorter duration of G1 phase correlated with both increased percentages of ATRi-induced ssDNA-positive cells (*P* = 0.003) and ATRi sensitivity (*P* = 0.002) (Figure 3B). These results strongly indicated that ATRi-induced ssDNA formation in LADC cells is a unique indicator of intrinsic replication stress that can predict ATRi sensitivity (38). Of the seven SMARCA4^MUT^ cell lines tested, six showed increases in the percentages of ATRi-induced ssDNA-positive cells, reaching over 10%, whereas five of the six SMARCA4^WT^ cell lines tested showed very little increase in the percentages of ATRi-induced ssDNA-positive cells, with none reaching 10% (Figure 3A), with these differences being statistically significant (Figure 3C, *P* = 0.005). Furthermore, a test for correlation between SMARCA4 status and ATRi sensitivity approached significance (Figure 3D, *P* = 0.073), although a few SMARCA4^WT^ cell lines (e.g. PC-9 and LC-OK cells) were comparable in ATRi sensitivity to SMARCA4^MUT^ cells. None of the previously reported biomarkers was associated with ATRi sensitivity of these LADC cell lines (Figure 1A and C; Supplementary Table S2). Thus, SMARCA4 deficiency causes intrinsic replication stress that is repressed by ATR activity in unperturbed cells. SMARCA4 deficiency also results in the accumulation of excessive ssDNA, which upon ATR inhibition by an ATRi induces RC.

We next assessed whether ATRi could inhibit SMARCA4-deficient tumors *in vivo* by treating A549 (SMARCA4^MUT^) and H1975 (SMARCA4^WT^) xenografted mice with ATRi or vehicle alone (Supplementary Figure S3E). We found that, compared with control treatment, ATRi had no effect on H1975 tumors but significantly inhibited the growth of A549 tumors (Figure 3E and F). To further evaluate the function of ATRi, these tumors were stained for Ki-67 and γH2AX. Ki-67 positivity was significantly lower in A549 than in H1975 cells, whereas γH2AX positivity was increased in A549 but not in H1975 cells (Figure 3G and H), suggesting that ATRi induced a greater extent of cell death in SMARCA4^MUT^ than in SMARCA4^WT^ cells *in vivo*, similar to results observed *in vitro*. Because absence of SMARCA4 expression was strongly associated with ATRi efficacy both *in vitro* and *in vivo*, these results provide a preclinical rationale for assessing SMARCA4 defects as a biomarker for ATRi.

### Loss of SMARCA4 increases replication stress and ATRi susceptibility

**Figure 4.**
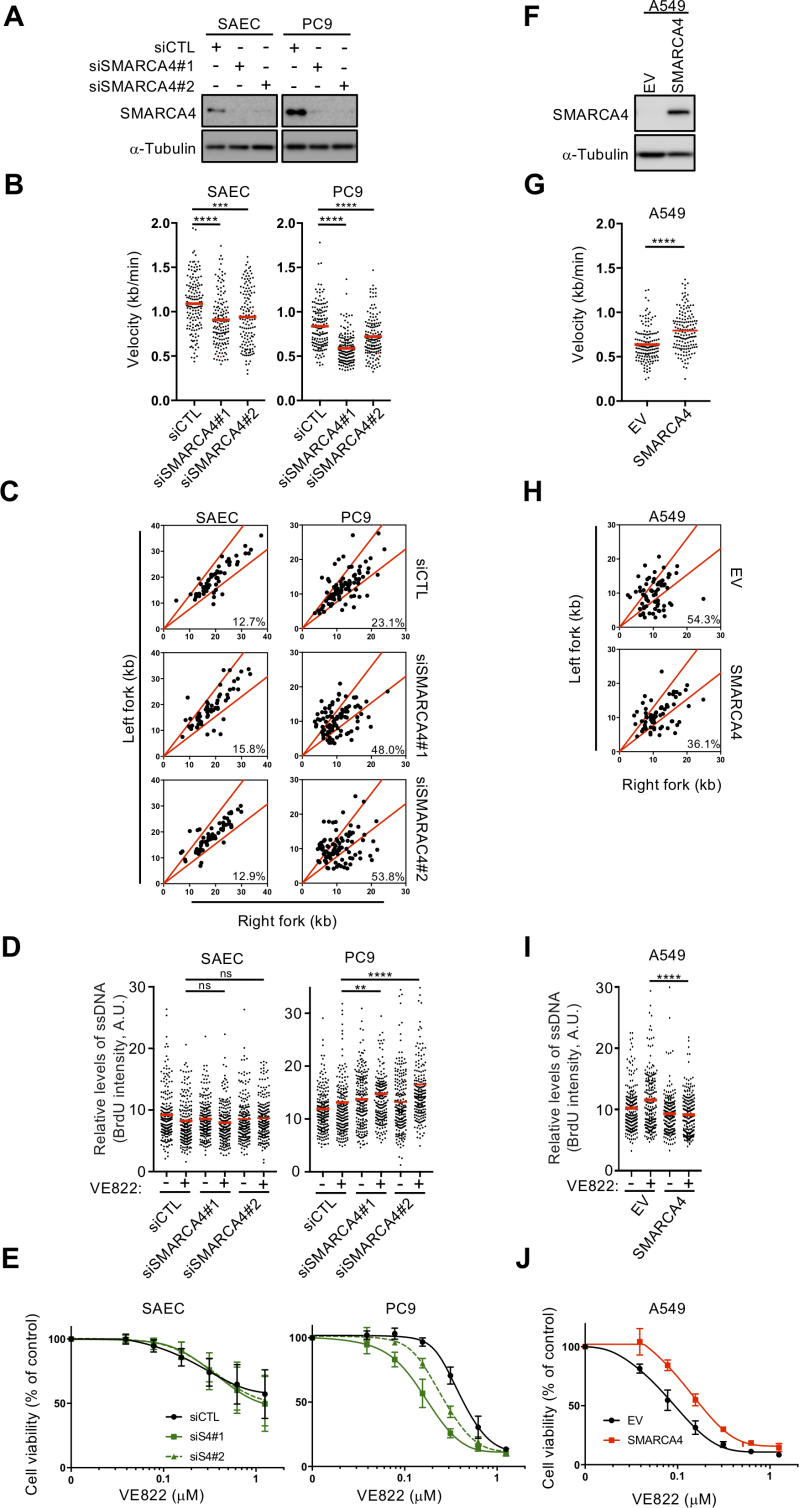
Loss of SMARCA4 increases intrinsic DNA replication stress and ATRi susceptibility. (**A**–**E**) SMARCA4^WT^ cells and SAECs were transfected with control siRNA (siCTL) or two independent SMARCA4 siRNAs. Forty-eight hours later, whole-cell lysates (WCLs) were prepared and the expression of SMARCA4 was analyzed by western blotting (**A**). Analysis of the velocities (**B**) and asymmetries (**C**) of individual forks by DNA fiber assays. Left- and right-moving sister fork (CldU tracks) lengths were measured and plotted. The central areas marked by red lines indicate sister forks with differences in length <30%. The percentages of each outlier, defined as asymmetric forks, are shown in each panel at bottom right (**C**). Representative results of two independent reproducible experiments are shown. (**D**) Quantification of the intensities of exposed ssDNA in 200 S-phase PC9 cells and SAECs transfected with siCTL or siSAMRCA4. Red bars represent the mean intensities. Representative results of two independent reproducible experiments. (**E**) Forty-eight hours after transfection with siCTL and siSMARCA4, the cells were re-seeded, incubated for 24 h and treated with varying doses of ATRi. Cell viability was measured using the PrestoBlue assay. The results represent the mean ± SD of three independent experiments. (**F**–**J**) SMARCA4^MUT^ cells were lentivirally transduced with SMARCA4 or empty vector (EV). WCLs were prepared and SMARCA4 levels were analyzed by western blotting (**F**). Analysis of the velocities (**G**) and asymmetry (**H**) of individual DNA fibers from A549 cells transduced with EV or SMARCA4, as described in (**B**) and (**C**). (**I**) Quantification of the ssDNA intensities of 200 S-phase A549 cells transduced with EV or SMARCA4 using the protocol described in (**D**). (**J**) A549 cells transduced with EV or SMARCA4 were treated with various concentrations of ATRi, and cell viability was measured by the PrestoBlue assay. The results represent the mean ± SD of three independent experiments.

The correlation between SMARCA4 status and replication stress in the panel of LADC cells suggested that loss of SMARCA4 elevates replication stress. To analyze the impact of SMARCA4 deficiency on the progression of individual replication forks, we measured the kinetics of DNA replication using a DNA fiber assay following sequential labeling with IdU and CldU. SMARCA4 depletion reduced the velocity of replication fork progression in SMARCA4^WT^ cells (PC9, H1975 and H2228) and normal SAECs (24) (Figure 4A and B; Supplementary Figure S4A and B). To identify stalled and collapsed forks, we analyzed the symmetry between the second CldU pulses from a fired origin in double-labeled DNA fibers. We measured the lengths of the CldU tracts for each pair of sister forks, plotted each pair as the right versus the left length and calculated the percentage of asymmetric forks. SMARCA4 depletion increased fork asymmetry >2-fold in the LADC cell lines PC9, H1975 and H2228, but had no effect in SAECs, suggesting that SMARCA4 protects stalled and collapsed forks specifically in LADC cells (Figure 4C and Supplementary Figure S4C). ATRi-induced ssDNA exposure was significantly higher, resulting in greater susceptibility to ATRi of SMARCA4-depleted LADC cells but not normal SAECs (Figure 4D and E; Supplementary Figure S4D and E). Interestingly, p53 and p21 increased in SMARCA4-depleted SAECs, suggesting that p21 induced by SMARCA4 depletion may decrease fork speed and minimize fork asymmetry in SAECs, as previously reported (48) (Figure 4B and C; Supplementary Figure S4F). Furthermore, depletion of p53 in SMARCA4-depleted SAECs did not induce p21, reduced fork speed and increased fork asymmetry and ATRi-induced ssDNA exposure. These findings suggest that a mechanism involving p53 and p21 can stop defective forks from further progression and limit replication stress in normal cells (Supplementary Figure S4G–I). By contrast, restoration of SMARCA4 expression decreased replication stress in SMARCA4 ^MUT^ cells (A427, A549 and H1299) as indicated by recovered replication fork velocity (Figure 4F and G; Supplementary Figure S4J and K) and a reduction of asymmetric fork progression compared with the empty virus-infected cells (Figure 4H and Supplementary Figure S4L). Similarly, ATRi-induced ssDNA exposure decreased in SMARCA4-restored LADC cells, followed by their reduced susceptibility to ATRi (Figure 4I and J; Supplementary Figure S4M and N). Collectively, these data suggest a model in which SMARCA4 plays a dominant role in regulating intrinsic replication stress and ATRi resistance in LADC cells independent of cell type.

### SMARCA4 loss causes ATRi-induced ssDNA exposure via Mre11-dependent degradation of nascent DNA at reversed replication forks

**Figure 5.**
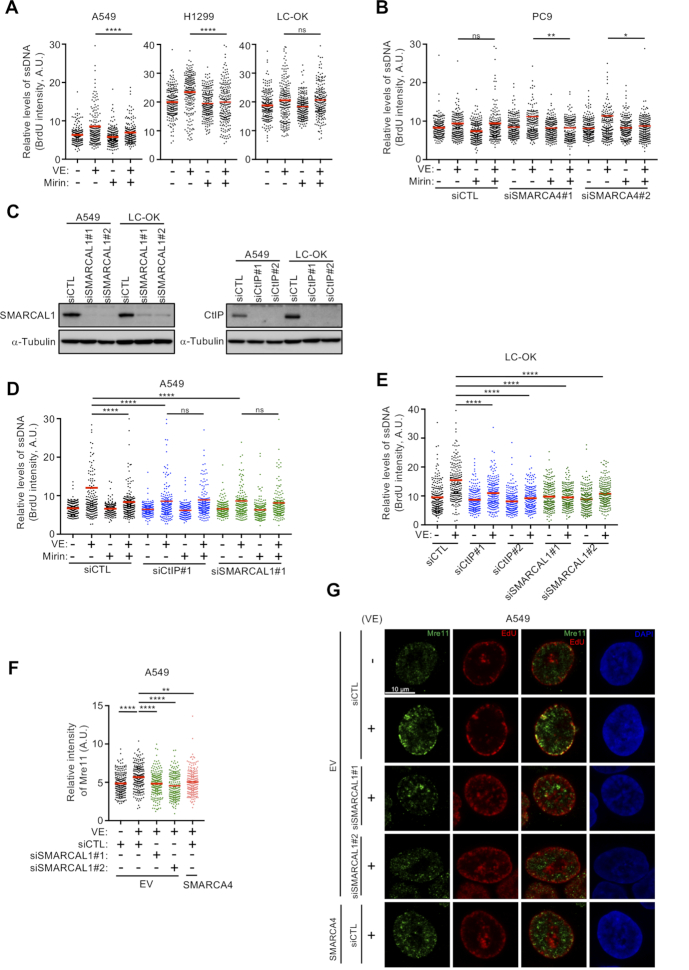
ATRi-induced ssDNA depends on replication fork reversal and Mre11 activity in SMARCA4-deficient cells. (**A**) SMARCA4^MUT^ (A549 and H1299) and SMARCA4^WT^ (LC-OK) cells were pretreated with the Mre11 inhibitor Mirin (50 μM) for 1 h, followed by treatment with ATRi (1 μM) for 2 h, and the exposed ssDNA intensities of 200 S-phase cells were quantified. Red bars represent the mean intensities. The results shown are representative of two independent reproducible experiments. (**B**) Analysis of exposed ssDNA in SMARCA4^WT^ PC9 cells transfected with siCTL or two independent SMARCA4 siRNAs for 48 h, pretreated or not with Mirin (1 h, 50 μM), followed by ATRi treatment (2 h, 1 μM). Quantification of the intensities of exposed ssDNA in 200 S-phase cells. Red bars represent mean intensities. The results shown are representative of two independent reproducible experiments. (**C**) SMARCA4^MUT^ A549 and SMARCA4^WT^ LC-OK cells were transfected with two independent SMARCAL1 or CtIP siRNAs for 48 h, and the levels of SMARCAL1 and CtIP were analyzed by western blotting. (**D**) Quantification of the intensities of exposed ssDNA in 200 S-phase A549 cells transfected with the indicated siRNAs and treated with ATRi and/or Mre11 inhibitor as in (**A**). Red bars represent the mean intensity. The results shown are representative of two independent reproducible experiments. (**E**) Quantification of the intensities of exposed ssDNA in 200 S-phase LC-OK cells transfected with the indicated siRNAs and treated with ATRi. Red bars represent the mean intensity. The results shown are representative of two independent reproducible experiments. (**F**, **G**) A549 cells transduced with EV or SMARCA4 were transfected with the indicated siRNAs. After 48 h, the cells were incubated with 10 μM EdU for 15 min and treated with 1 μM ATRi for 2 h. (**F**) Quantification of the intensities of total Mre11 in 200 S-phase cells. Red bars represent the mean intensities. The results shown are representative of two independent reproducible experiments. (**G**) Representative images of Mre11 and EdU staining of indicated cells. The nuclei were counterstained with DAPI. Scale bar: 10 μm.

Because RCs are strongly induced by ATRi treatment, especially in SMARCA4 ^MUT^ cells (Figure 2A and B), we assessed whether inhibition of ATR activity results in the progressive destabilization of replication forks in these cells (46). Mre11 was shown to mediate replication fork degradation in cell lines with mutations in HR factors, including BRCA1, BRCA2 and Rad51 (49–51). We therefore assessed whether SMARCA4-deficient LADC cells show similar Mre11-dependent degradation of nascent replication tracts and examined the levels of expression of HR factors. Surprisingly, Mirin, an Mre11 inhibitor, suppressed ATRi-induced ssDNA exposure in SMARCA4^MUT^ (A549 and H1299) but not in SMARCA4^WT^ (LC-OK) cells, suggesting that replication forks are not protected from Mre11-mediated degradation in SMARCA4-deficient cells (Figure 5A), even in the presence of HR-associated factors (Supplementary Figure S5A). Mirin also specifically suppressed the ATRi-induced ssDNA exposure exacerbated by SMARCA4 depletion in PC9 and H1975 cells, but not in control-depleted cells (Figure 5B and Supplementary Figure S5B). To test whether reversed replication forks are required for this fork degradation, we analyzed the ATRi-induced ssDNA levels in SMARCAL1 (SWI/SNF-related, matrix-associated, actin-dependent regulator of chromatin, subfamily A-like 1)-depleted cells. Consistent with previous results (52), we found that SMARCAL1 depletion appreciably limited ATRi-induced ssDNA exposure and that Mirin did not further reduce ATRi-induced ssDNA exposure, suggesting that replication fork reversal is a prerequisite for fork degradation (Figure 5C and D; Supplementary Figure S5C). We also evaluated whether CtIP, which interacts with the Mre11 nuclease that initiates Mre11-mediated degradation of stalled forks (53,54), is required for ATRi-induced ssDNA exposure. CtIP depletion in SMARCA4^MUT^ cells largely suppressed ATRi-induced ssDNA exposure, with Mirin having no additive effect on this phenotype, suggesting that attack by Mre11 requires CtIP-mediated nucleolytic initiation at reversed forks (Figure 5C and D; Supplementary Figure S5C). In SMARCA4^WT^ cells (LC-OK), depletion of both SMARCAL1 and CtIP suppressed ATRi-induced ssDNA exposure, suggesting that even in SMARCA4-proficient cells, CtIP-mediated nucleolytic attack at reversed forks might be a target of alternative nucleases (Figure 5E). To investigate how ATRi-induced double-strand breaks (DSBs) associated with RC are generated at reversed forks in SMARCA4^MUT^ cells, we compared the effects of Mre11 on ATRi- and CPT-induced γH2AX. ATRi increased the fraction of γH2AX-positive cells via a process that was largely inhibited by Mirin, whereas CPT strongly induced the formation of γH2AX-positive cells via a process that was not inhibited by Mirin (Supplementary Figure S5D). Moreover, knockdown of SLX4, which orchestrates the Holliday junction endonucleases involved in DSB generation at stalled forks, did not affect ATRi-induced ssDNA exposure in SMARCA4^MUT^ cells (Supplementary Figure S5E and F), suggesting that ATRi induces Mre11-dependent ssDNA exposure followed by the formation of DSBs that lead to fork collapse. Furthermore, inhibition of ATR activity resulted in recruitment of Mre11 to SMARCA4^MUT^ (A549 and H1299) cells and SMARCA4-depleted (PC9) cells, but not to SMARCA4-restored cells and control-depleted cells (Figure 5F; Supplementary Figure S5G and H), with Mre11 recruitment being dependent on SMARCAL1 (Figure 5F and Supplementary Figure S5G). Mre11 colocalized with nascent DNA labeled for a short time (15 min) with EdU immediately before ATRi treatment. These colocalizations were found mainly but were not limited to cells in late S phase (Figure 5G and Supplementary Figure S5I), in which the heterochromatic region at the nuclear periphery is replicated, in a manner dependent on SMARCAL1 (Figure 5G). Together, these results suggest that, in the absence of ATR regulation, Mre11 might attack double-stranded DNA, consisting of reannealed nascent strands produced at regressed forks in SMARCA4-deficient cells.

### SMARCA4 loss increases heterochromatin formation

**Figure 6.**
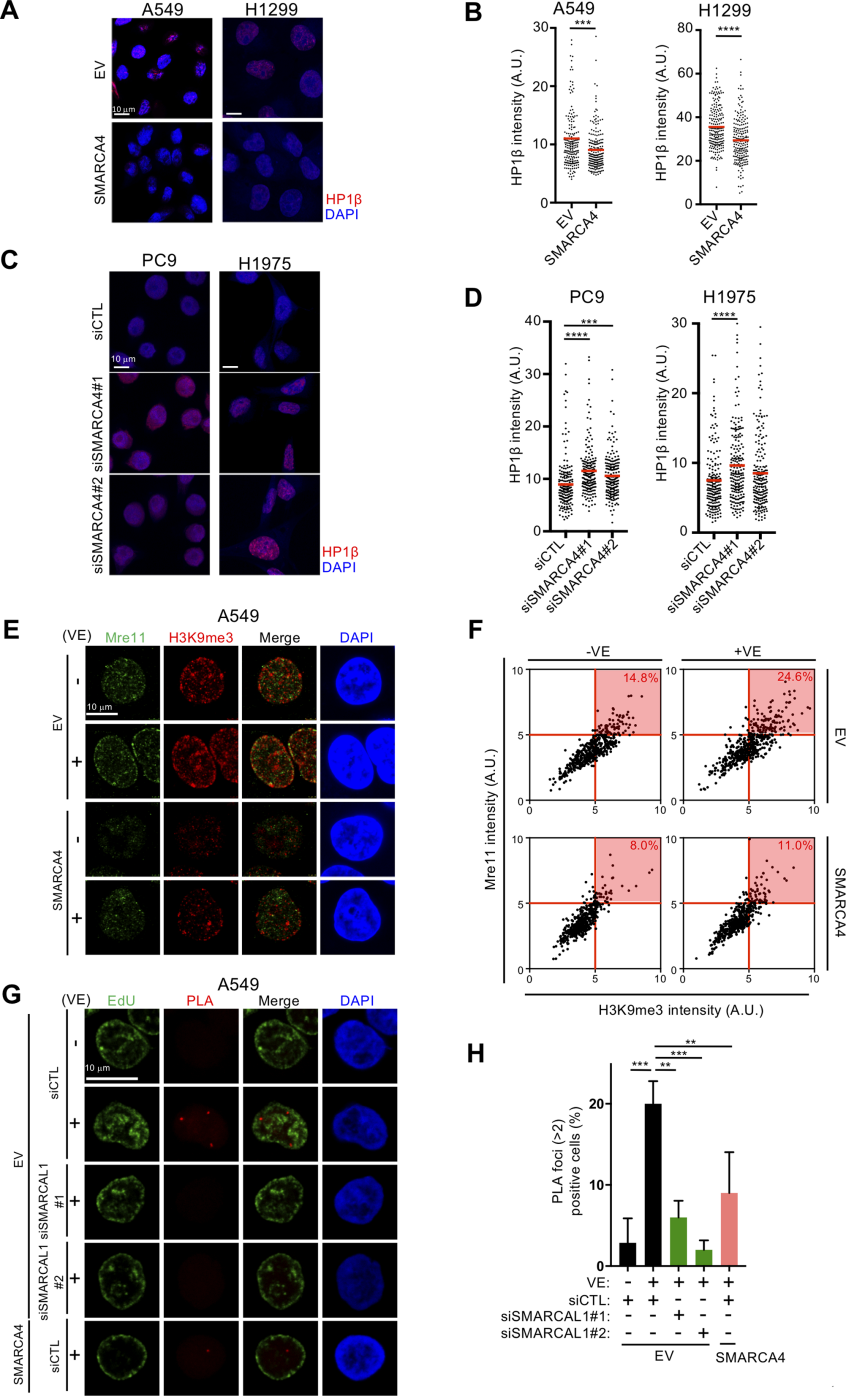
Increased heterochromatin formation induced by SMARCA4 loss promotes ATRi-induced fork reversal and Mre11 recruitment. (**A**) SMARCA4^MUT^ (A549 and H1299) cells transduced with EV or SMARCA4 were immunostained with anti-HP1β antibody, and the nuclei were counterstained with DAPI. Scale bar: 10 μm. (**B**) Quantification of the intensities of individual nuclei in (**A**). Red bars represent mean intensities. Representative results of two independent reproducible experiments are shown. (**C**) SMARCA4^WT^ (PC9 and H1975) cells were transfected with siCTL or siSMARCA4 for 48 h and immunostained with anti-HP1β antibody. The nuclei were counterstained with DAPI. Scale bar: 10 μm. (**D**) Quantification of the intensities of total HP1β intensities in individual nuclei in (**C**). Red bars represent mean intensities. Representative results of two independent reproducible experiments are shown. (**E**) A549 cells transduced with EV or SMARCA4 were treated with 1 μM ATRi for 2 h and immunostained with anti-H3K9me3 and anti-Mre11 antibody, and the nuclei were counterstained with DAPI. Scale bar: 10 μm. (**F**) Scatter diagram showing the total intensity of H3K9me3 and Mre11 in (**E**). Percentages of cells doubly positive for Mre11 and H3K9me3 (red) are indicated. Results are representative of two independent reproducible experiments. (**G**) A549 cells transduced with EV or SMARCA4 were transfected with the indicated siRNAs. After 48 h, the cells were incubated with 10 μM EdU for 15 min and treated with 1 μM ATRi for 2 h. After EdU staining, PLAs using anti-HP1α and anti-SMARCAL1 antibodies were performed. The nuclei were counterstained with DAPI. Scale bar: 10 μm. (**H**) Quantification of PLA signal in (**G**). The results represent the mean ± SD of three independent experiments.

**Figure 7.**
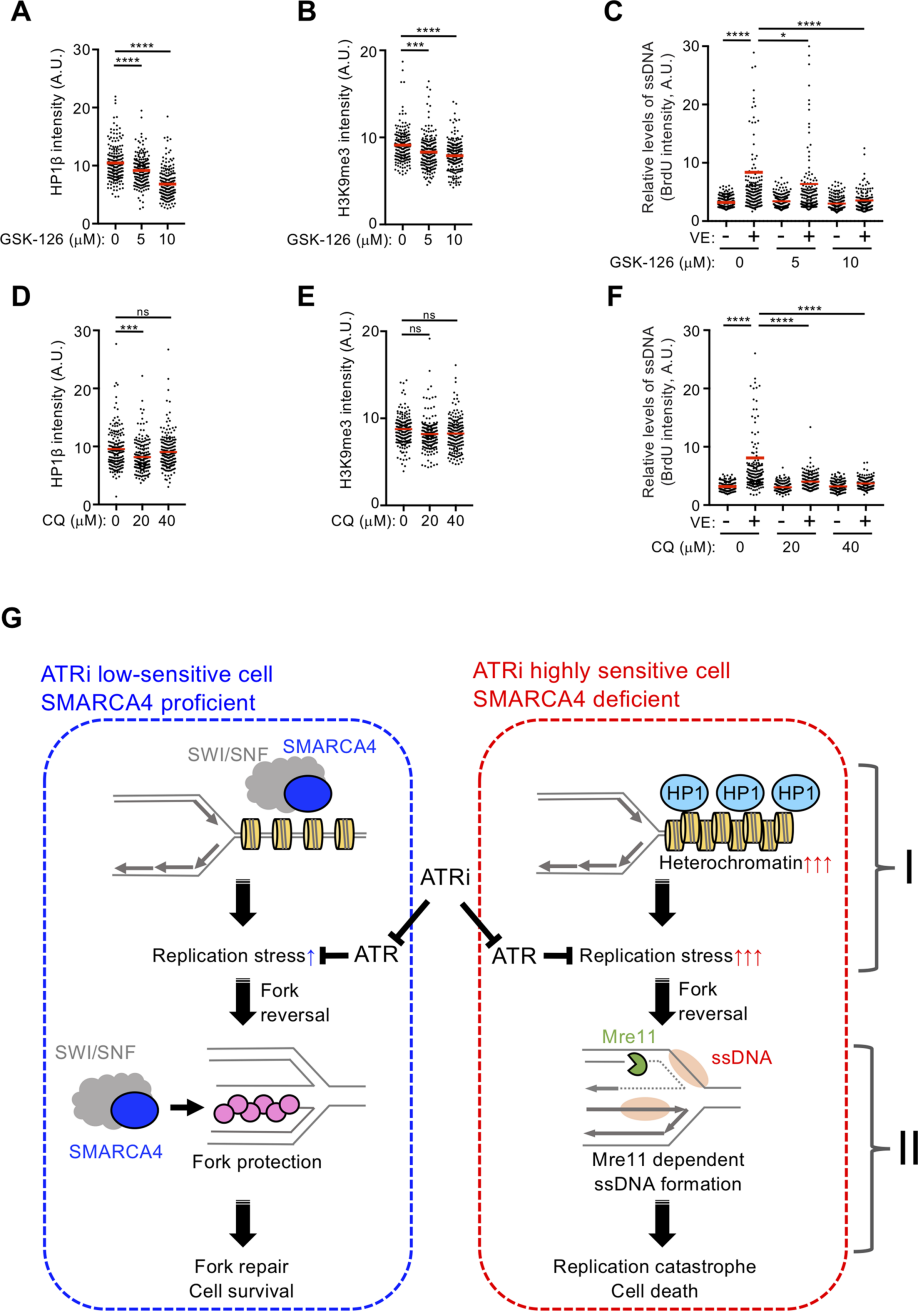
Resolving heterochromatin reduces DNA replication stress in SMARCA4-deficient cells. SMARCA4^MUT^ (A549) cells were pretreated with the indicated concentrations of GSK-126 for 48 h and immunostained with anti-HP1β (**A**) or anti-H3K9me3 (**B**) antibody, followed by DAPI counterstaining of the nuclei. The intensities of individual nuclei are quantified, with the red bars representing mean intensities. Results representative of two independent reproducible experiments are shown. (**C**) SMARCA4^MUT^ (A549) cells were pretreated with the indicated concentrations of GSK-126 for 46 h, followed by treatment with ATRi (1 μM) for 2 h, and the exposed ssDNA intensities of 200 S-phase cells were quantified. Red bars represent the mean intensities. The results shown are representative of two independent reproducible experiments. SMARCA4^MUT^ (A549) cells were pretreated with the indicated concentrations of CQ for 4 h and immunostained with anti-HP1β (**D**) or anti-H3K9me3 (**E**) antibody, and the nuclei were counterstained with DAPI. The intensities of individual nuclei were quantified, with red bars representing the mean intensities. Representative results of two independent reproducible experiments are shown. (**F**) SMARCA4^MUT^ (A549) cells were pretreated with the indicated concentrations of CQ for 2 h, followed by treatment with ATRi (1 μM) for 2 h, and the exposed ssDNA intensities of 200 S-phase cells were quantified. Red bars represent the mean intensities. The results shown are representative of two independent reproducible experiments. (**G**) Proposed model explaining the increased ATRi susceptibility in SMARCA4-deficient cells. Loss of SMARCA4 results in (I) accumulation of difficult-to-replicate sites, such as heterochromatin, and (II) defects in the protection of reversed replication forks from nucleolytic attack by Mre11. These mechanisms lead to increased ssDNA exposure and subsequent cell death due to RC.

The nuclear periphery is a functional compartment enriched in heterochromatin, a replication fork barrier that may reduce fork velocity and stall the replication machinery (55). The finding, that ATRi induced colocalization of Mre11 and nascent DNA at the nuclear periphery, prompted an examination of heterochromatin induction in SMARCA4-deficient cells. Restoration of SMARCA4 in SMARCA4^MUT^ cells (A549 and H1299) reduced the number and overall intensity of HP1β foci (Figure 6A and B), whereas depletion of SMARCA4 in SMARCA4^WT^ cells (PC9 and H1975) increased the number and overall intensity of HP1β foci (Figure 6C and D). By contrast, depletion of SMARCA2, a mutually exclusive catalytic ATPase subunit present in SWI/SNF complexes, did not increase the overall intensity of HP1β foci and ATRi-induced ssDNA exposure (Supplementary Figure S6A–C). These results suggest that the loss of SMARCA4 specifically leads to increased facultative heterochromatin formation, increasing intrinsic replication stress under unperturbed conditions in LADC cells (Figure 4B–D and G–I). We also observed increased heterochromatin formation in SAECs, in which depletion of SMARCA4 did not increase fork asymmetry, ATRi-induced ssDNA exposure and ATRi sensitivity (Supplementary Figure S6D). These findings suggest that SMARCA4 loss-associated heterochromatin may activate p53 and p21 to minimize replication stress in normal SAECs (Supplementary Figure S4F–I). We also analyzed whether Mre11 accumulates in the vicinity of hard-to-replicate heterochromatin regions, where replication forks might stall. SMARCA4^MUT^ cells (A549) showed greater accumulation of trimethylated lysine 9 on histone H3 (H3K9me3), a marker of heterochromatin-associated histone, than SMARCA4-restored cells (Figure 6E and F). Depletion of SMARCAL1, which reduced ATRi-induced Mre11 accumulation (Figure 5G), did not reduce H3K9me3, suggesting that heterochromatin itself is not sufficient and that a fork reversal is required for ATRi-induced accumulation of Mre11 and subsequent ssDNA exposure (Supplementary Figure S6E). ATRi-induced Mre11 foci of SMARCA4^MUT^ cells in late S phase juxtaposed with H3K9me3 foci at the nuclear periphery (Figure 6E), and ATRi treatment increased the number of cells doubly positive for Mre11 and H3K9me3 (24.6%) when compared with untreated cells (14.8%) (Figure 6F), strongly suggesting that Mre11 attacks stalled and regressed replication forks around the heterochromatic region. Next, to determine whether, in the absence of SMARCA4, replication fork reversal in heterochromatic regions gives rise to structures in which Mre11 is recruited to generate ATRi-induced ssDNA, we performed PLA using antibodies recognizing SMARCAL1 and HP1α, a fundamental unit of heterochromatin packaging. In SMARCA4-deficient cells, ATR inhibition increased PLA foci, which were abolished by depletion of SMARCAL1 using two independent SMARCAL1 siRNAs, suggesting that SMARCAL1 is recruited to heterochromatin upon ATR inhibition (Figure 6G and H). Consistent with Mre11 foci induced by ATRi, ATRi-induced PLA foci gradually increased during S phase, suggesting that SMARCA4 loss causes replication stress mainly in but not limited to late S phase (Supplementary Figures S5I and S6F). By contrast, ATR inhibition did not increase PLA foci in the SMARCA4-restored cells (Figure 6G and H), which harbor decreased heterochromatin (Figure 6A). These results suggest that, when ATR is inhibited, SMARCAL1 promotes fork reversal, in which replication forks encounter hard-to-replicate heterochromatic regions in SMARCA4-deficient cells. Finally, we assessed whether inhibition of the PRC complex, which resolves heterochromatin by overcoming histone modifications, could reduce replication stress. GSK-126, an inhibitor of EZH2 in the PRC2 complex (56), dose dependently reduced the number and overall intensity of both HP1β foci and H3K9me3 (Figure 7A and B), resulting in reduced ATRi-induced ssDNA exposure (Figure 7C) in SMARCA4-deficient A549 cells. However, depletion of HP1β did not reduce the overall intensity of H3K9me3 and ATRi-induced ssDNA exposure (Supplementary Figure S7A–C). Remarkably, CQ (57,58), a DNA intercalating drug that opens chromatin, reduced ATRi-induced ssDNA exposure although it did not reduce HP1β foci and H3K9me3 (Figure 7D–F). These results suggest that the physically closed chromatin structure present in heterochromatin hinders replication fork progression and causes replication stress.

## DISCUSSION

Because LADC cells, as well as several other cancer types, frequently carry homozygous SMARCA4-inactivating mutations (9,10,42,59–62), the identification of vulnerabilities caused by loss of SMARCA4 expression that result in synthetic lethal relationships may be useful for developing treatment strategies for this type of cancer. Our results indicate that loss of SMARCA4 *in vitro* and *in vivo* enhances intrinsic DNA replication stress and sensitizes cells to ATRi without the need for extrinsic DNA replication stress induced by any other drugs. In this context, the absence of SMARCA4 drives the formation of facultative heterochromatin, a difficult-to-replicate structure, whereas SMARCAL1 promotes fork reversal and CtIP initiates nucleolytic processing as prerequisites for ATRi-induced ssDNA exposure, an indicator of intrinsic replication stress (38). These findings suggest that heterochromatin is a cause of intrinsic replication stress in SMARCA4-deficient cells. Intriguingly, we found that an Mre11 inhibitor suppressed ATRi-induced ssDNA exposure only in the absence of SMARCA4, indicating that SMARCA4 protects replication forks against nucleolytic attack on nascent DNA by Mre11 when ATR activity is inhibited. Therefore, loss of SMARCA4 confers ATRi susceptibility via at least two distinct mechanisms, by increasing replication stress via heterochromatin formation (Figure 7G, I) and by destabilizing reversed replication forks (Figure 7G, II).

### Loss of SMARCA4 increases intrinsic DNA replication stress and predicts ATRi susceptibility

Preclinical studies found that ATRis induced both chemo- and radiosensitization (63–69). This led to ongoing clinical studies, most of which are testing ATRis in combination with DNA cross-linking agents, topoisomerase I or II inhibitors, and radiation. ATRis are expected to potentiate the activity of cytotoxic DNA-damaging agents and radiotherapy by inhibiting ATR-dependent DDR, which orchestrates DNA repair and other important processes. Consistent with previous reports, our results indicate that synergy between the effects of ATRi and cisplatin dramatically promoted the death of SMARCA4^WT^ cells and to some extent of SMARCA4^MUT^ cells. In addition, treatment with ATRi alone was sufficient to induce a high level of ATRi-induced ssDNA exposure and RC-associated DNA damage, as shown by pan-nuclear γH2AX staining, in SMARCA4^MUT^ cells, whereas co-treatment with cisplatin further increased the fraction of pan-nuclear γH2AX-positive cells. Although cisplatin did not increase the fraction of ssDNA-positive cells in all of the tested LADC cell lines, it induced discrete γH2AX foci. Treatment with ATRi and cisplatin increased the fraction of pan-nuclear γH2AX-positive cells, even in SMARCA4^WT^ cells, suggesting that cisplatin-induced replication stress renders cells highly susceptible to ATRi in conjunction with the ATRi-mediated abrogation of cisplatin-induced DDR. Ranking of these cell lines by the fraction of ATRi-induced ssDNA-positive cells showed that their ATRi sensitivities were clearly correlated, with SMARCA4^MUT^ cells showing greater replication stress and ATRi sensitivity than SMARCA4^WT^ cells. Consistent with these *in vitro* experiments, treatment with ATRi significantly inhibited SMARCA4^MUT^ but not SMARCA4^WT^ tumors *in vivo*, strongly suggesting that the level of replication stress is critical for ATRi susceptibility and that SMARCA4 deficiency may be a promising biomarker for predicting the sensitivity of LADC cells to ATR inhibition.

### Loss of SMARCA4 enhances heterochromatin formation leading to replication stress

How does loss of SMARCA4 increase replication stress? The BAF (SWI/SNF) complex, in which SMARCA4 is a constitutive factor, opposes PRCs via rapid, ATP-dependent eviction, leading to the formation of accessible chromatin; furthermore, reversal of this process results in reassembly of facultative heterochromatin on a minute-by-minute basis (4). BAF removal from activated promoters also permits the recruitment of the HP1/Suv39h1 heterochromatin complex to deposit H3K9me3 domains, resulting in gene silencing (70). Indeed, our results showed that, in the absence of SMARCA4 (i.e. in SMARCA4^MUT^ and SMARCA4-depleted SMARCA4^WT^ cells), the number and intensity of HP1β and H3K9me3 foci were greater than in cells containing SMARCA4 (i.e. SMARCA4-rescued SMARCA4^MUT^ and SMARCA4^WT^ cells). These results suggest that loss of SMARCA4 is necessary and sufficient to drive facultative heterochromatin formation in LADC cells. Moreover, cells lacking SMARCA4 showed a higher incidence of stalled replication forks and increased ATRi-induced ssDNA exposure, leading to greater ATRi sensitivity of each isogenic counterpart. Interestingly, resolving heterochromatin not only by the EZH2 inhibitor but also by CQ reduced ATRi-induced ssDNA exposure. This finding suggested that a closed chromatin environment in SMARCA4-defecient LADC cells caused replication stress, whereas PRC-dependent silencing histone modifications and HP1 recruitment did not.

An alternative pathway may also be involved in the elevation of intrinsic replication stress in SMARCA4-deficient cells. Oxygen consumption, respiratory capacity and ROS levels are elevated in SMARCA4 mutant cells, resulting in a dependence on oxidative phosphorylation, which can be targeted by OXPHOS inhibitors (71). Cells lacking ARID1A, another core component of SWI/SNF complexes, have a low basal level of the antioxidant glutathione and higher ROS levels (72). Because elevated ROS levels induce slow replication fork progression (73,74), loss of SMARCA4 could enhance intrinsic replication stress by elevating ROS levels. Furthermore, SMARCA4 colocalizes with DNA replication factors (24), and recruits topoisomerases I (75) and II (23) to chromatin, suggesting that SMARCA4-deficient cells may experience DNA replication stress resulting from failed decatenation of supercoiled DNA. Thus, loss of SMARCA4 or defective SWI/SNF complexes could enhance intrinsic replication stress via multiple pathways.

### Loss of SMARCA4 fails to block Mre11-mediated destabilization of reversed replication forks in heterochromatic regions

Replication fork reversal is a fork-stabilizing structure that protects against fork stalling. Several proteins, including SMARCAL1, ZRANB3 and HTLF, catalyze the formation of reversed replication forks (76). In particular, SMARCAL1 is required to address endogenous sources of replication stress in the absence of exogenous drugs (77–79). ATR phosphorylates SMARCAL1 on Ser652 to negatively regulate its fork remodeling activity, indicating that ATRi promotes replication fork reversal (80). Furthermore, CtIP exhibits an intrinsic endonuclease activity toward DNA secondary structures in protecting common fragile sites (81), and initiates Mre11-dependent and/or -independent degradation of stalled forks (53,81). We found that ATRi-induced ssDNA exposure was suppressed in SMARCAL1- or CtIP-depleted cells irrespective of their SMARCA4 status, suggesting that ATRi-induced ssDNA exposure may largely depend on fork reversal and processing. Unexpectedly, however, we found that ATRi-induced ssDNA exposure was suppressed by Mre11 inhibition only in the absence of SMARCA4, and that Mre11 inhibition did not additively reduce ATRi-induced ssDNA exposure in SMARCAL1- and CtIP-depleted cells. These observations suggest that Mre11 plays a critical role downstream of CtIP at SMARCAL1-mediated reversed replication forks in SMARCA4-deficient cells. The mechanisms underlying ATRi-induced ssDNA exposure in SMARCA4-expressing cells remain to be elucidated but most likely involve alternative nucleases. In the absence of SMARCA4, the reduction in the number of ATRi-induced ssDNA-positive cells after Mre11 inhibition coincides with a reduced number of γH2AX-positive cells. By contrast, the CPT-dependent increase in the number of γH2AX-positive cells was insensitive to Mre11 inhibition under our experimental conditions. These findings suggest that, in unperturbed, ATR-inactivated SMARCA4-deficient cells, Mre11 attacks nascent DNA at regressed replication forks before the appearance of SLX4-generated DSBs (80). Moreover, treatment of SMARCA4^MUT^ cells with ATRi resulted in the colocalization of Mre11 foci with nascent DNA, with these Mre11 foci juxtaposed to heterochromatic H3K9me3 foci at the nuclear periphery in late S-phase cells. PLAs using antibodies to SMARCAL1 and HP1α further confirmed that SMARCAL1 is recruited to heterochromatin in SMARCA4^MUT^ cells treated with ATRi. Taken together, our findings indicate that SMARCA4 plays a crucial role in protecting intrinsic replication stress-induced stalled and reversed replication forks from Mre11-dependent fork destabilization in heterochromatic regions. This SMARCA4-dependent fork protection mechanism is distinct from the mechanisms by which other HR factors protect replication forks in the presence of DNA-damaging agents. Furthermore, our findings suggest that ATR enhances fork stability and cell survival in the face of dramatic increases in intrinsic replication stress stemming from loss of SMARCA4.

### Targeting SMARCA4-deficient cancers with ATRis

Our study demonstrates that a synthetic lethal interaction between loss of SMARCA4 and ATR inhibition represents a vulnerability in LADC that could be therapeutically exploited. LADC patients carrying SMARCA4 mutations are distinct from patients with putative driver oncogenes (e.g. EGFR mutations), for whom molecular targeted therapies are available. Our findings suggest that patients with SMARCA4-deficient LADC may benefit from treatment strategies that include ATRis. Because SMARCA4 deficiency is common in small cell ovarian cancer (82), Burkitt lymphoma (83) and pediatric medulloblastoma (84), among other tumor types (85), ATRi monotherapy may be effective and specific against other SMARCA4-deficient cancer cells with minimal undesirable effects.

## Supplementary Material

zcaa005_Supplemental_File
