# Supplementary material for: SMARCA4 deficiency-associated heterochromatin induces intrinsic DNA replication stress and susceptibility to ATR inhibition in lung adenocarcinoma
Source: NAR Cancer. 2020 May 1;2(2):zcaa005. doi: 10.1093/narcan/zcaa005 (PMC8210217; doi:10.1093/narcan/zcaa005)
Supplement: zcaa005_Supplemental_File [file narcancer_2_2_zcaa005_s8.pdf]

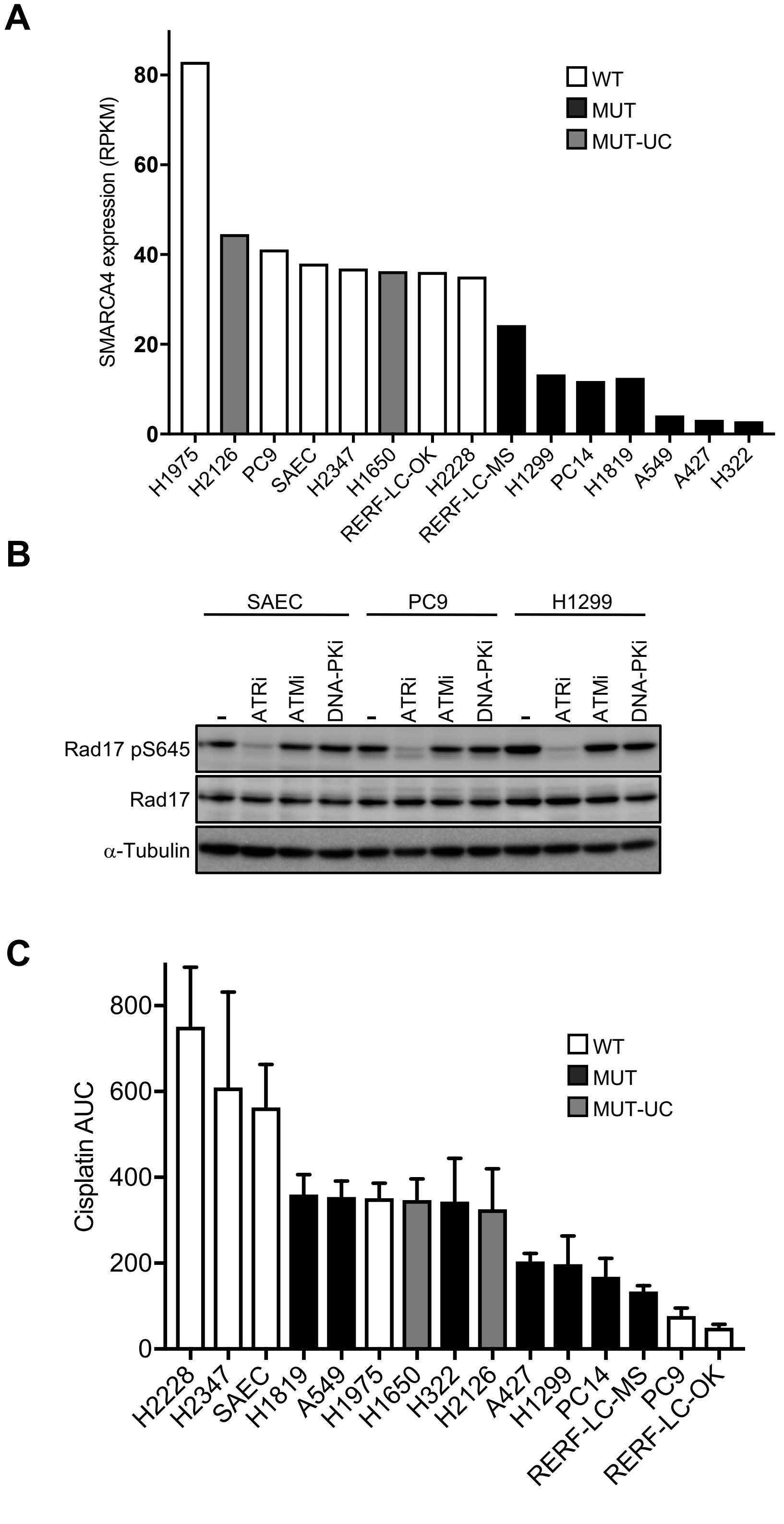

Supplementary Figure S1. SMARCA4 deficiency is not associated with cisplatin sensitivity.

(**A**) SMARCA4 mRNA expression levels of SAECs and LADC cells. (**B**) SAECs, H1299 and PC9 cells were treated with DMSO (-), ATRi (VE822, 1 μM), ATMi (KU55933, 10 μM) or NU7441 (DNA-PKi, 10 μM) for 2 h. The pRad17 levels were analyzed via western blotting. (**C**) SAECs and LADC cells expressing $SMARCA4^{WT}$ (white), $SMARCA4^{MUT}$ (black), $SMARCA4^{MUT\text{-}UC}$ (gray) were treated with increasing cisplatin doses (0.08–10 μM) for 6 days. Cell viability was measured via the PrestBlue assay. The area under the curve (AUC) values of each cell line are arranged in order. WT; wild type, MUT; deleterious mutation and MUT-UC; uncharacterized mutation. The values represent the mean $\pm$ S.D. of three independent experiments.

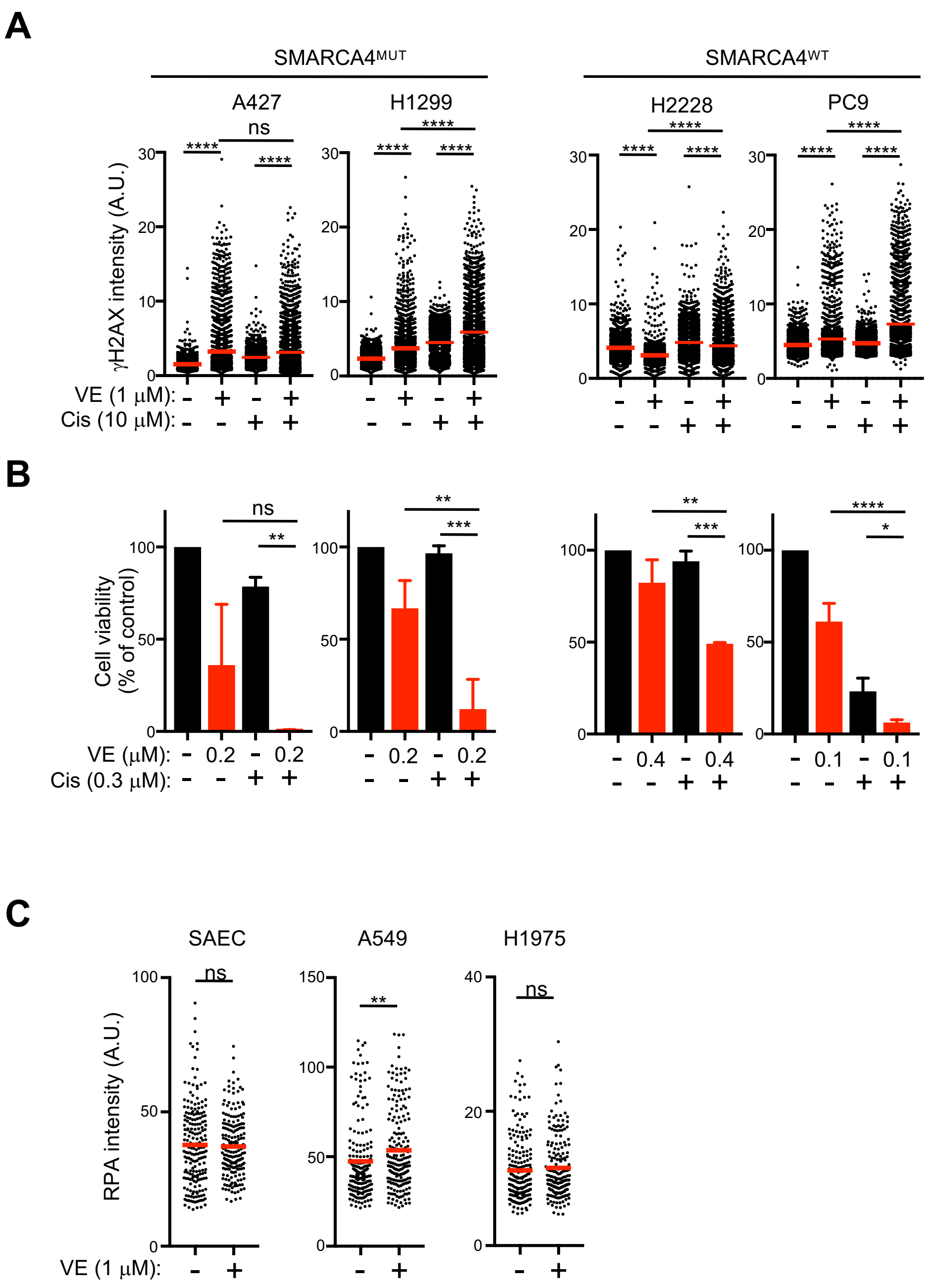

Supplementary Figure S2. Cisplatin-mediated DNA replication stress synergistically enhances ATRi-induced replication catastrophe.

(**A**) Quantification of the γH2AX intensities of 2,000 cells treated with ATRi and cisplatin is shown. Red bars represent the mean intensities. Representative results of two independent reproducible experiments are shown. (**B**) Cells were treated with ATRi and/or cisplatin at the indicated doses for 6 days and analyzed via the PrestBlue assay. The values represent the mean ±S.D. of three independent experiments. (**C**) Quantification of the RPA intensities of 200 S-phase cells is shown. Red bars represent the mean intensities. Representative results of two independent reproducible experiments are shown.

Kurashima et al. Supplementary Figure S2

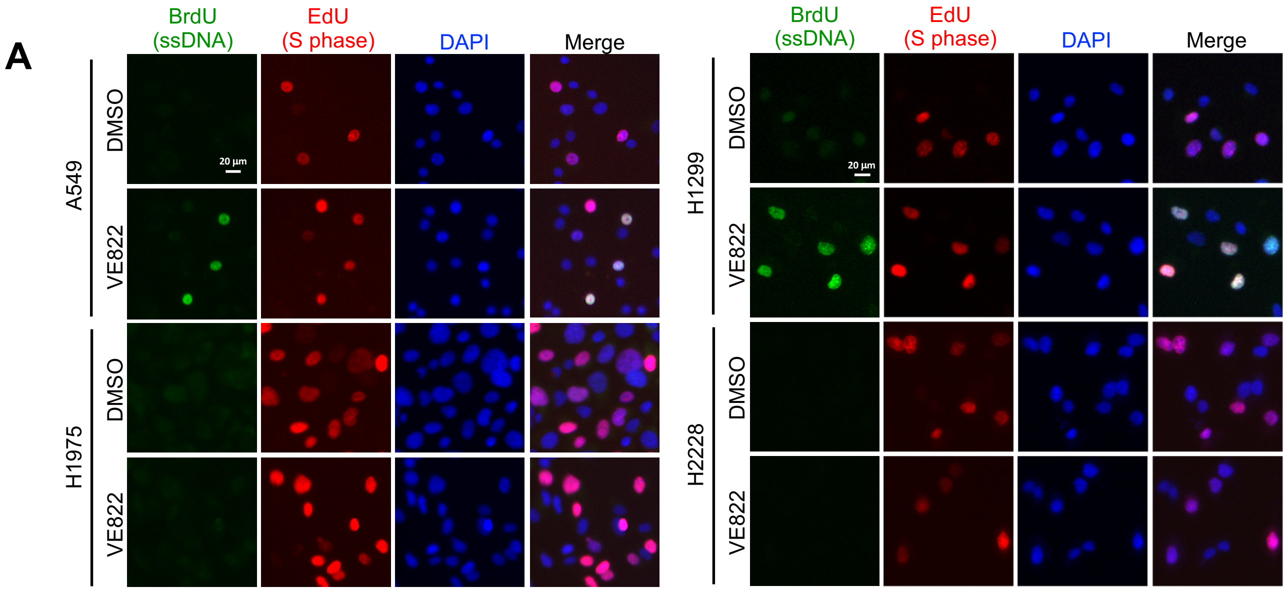

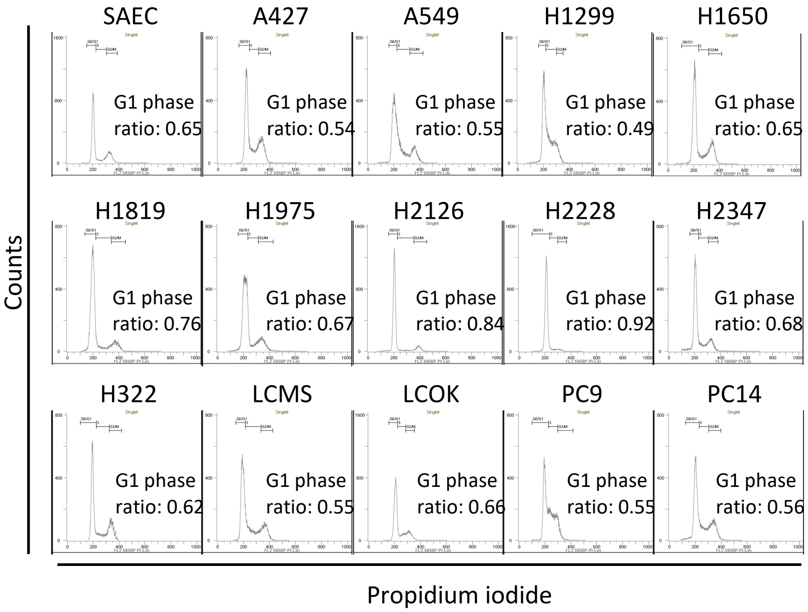

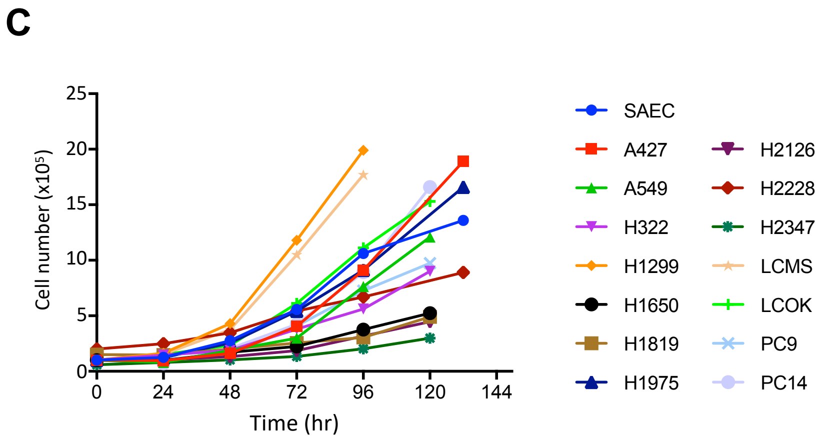

D

Cell line	Doubling time(h)	G1 phase ratio	G1 phase duration (h)
SAEC	23	0.65	14.7
A427	21	0.54	11.1
A549	19	0.55	10.5
H1299	16	0.49	7.9
H1650	31	0.65	20.1
H1819	36	0.76	27.4
H1975	24	0.68	16.1
H2126	41	0.84	33.8
H2228	36	0.92	33.0
H2347	41	0.68	27.9
H322	25	0.62	15.3
LCMS	16	0.55	8.9
LCOK	22	0.66	14.6
PC9	22	0.55	11.8
PC14	22	0.56	12.4

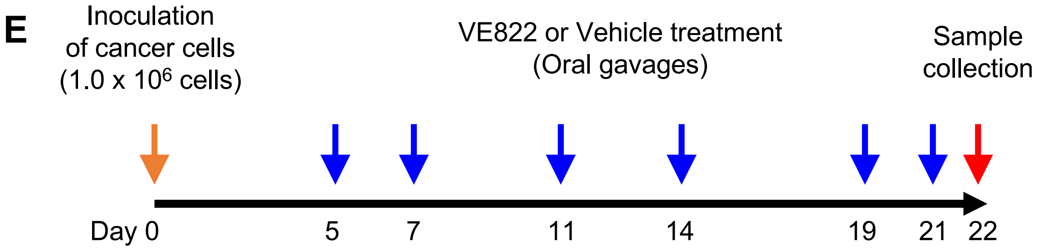

Kurashima et al. Supplementary Figure S3

Supplementary Figure S3. Cell cycle profiles and growth curves of SAECs and LADC cells.

(**A**) Representative images of exposed ssDNA in indicated cells as described in Figure 3A. Scale bars: 20 µm. (**B**) Cell cycle profiles of SAECs and LADC cells. Exponentially growing cells were fixed overnight in 70% Ethanol at -30 ºC and then stained with propidium iodide. The DNA content of the cells was analyzed via flow cytometry. The G1 phase ratios are shown. Representative results of two independent reproducible experiments are shown. (**C**) Cell proliferation growth curves of SAECs and LADC cells. Representative results of two independent reproducible experiments are shown. (**D**) G1 phase duration was estimated via the doubling time multiplied by the G1 phase ratio (see Methods). (**E**) Schematic schedule of mouse xenograft model.

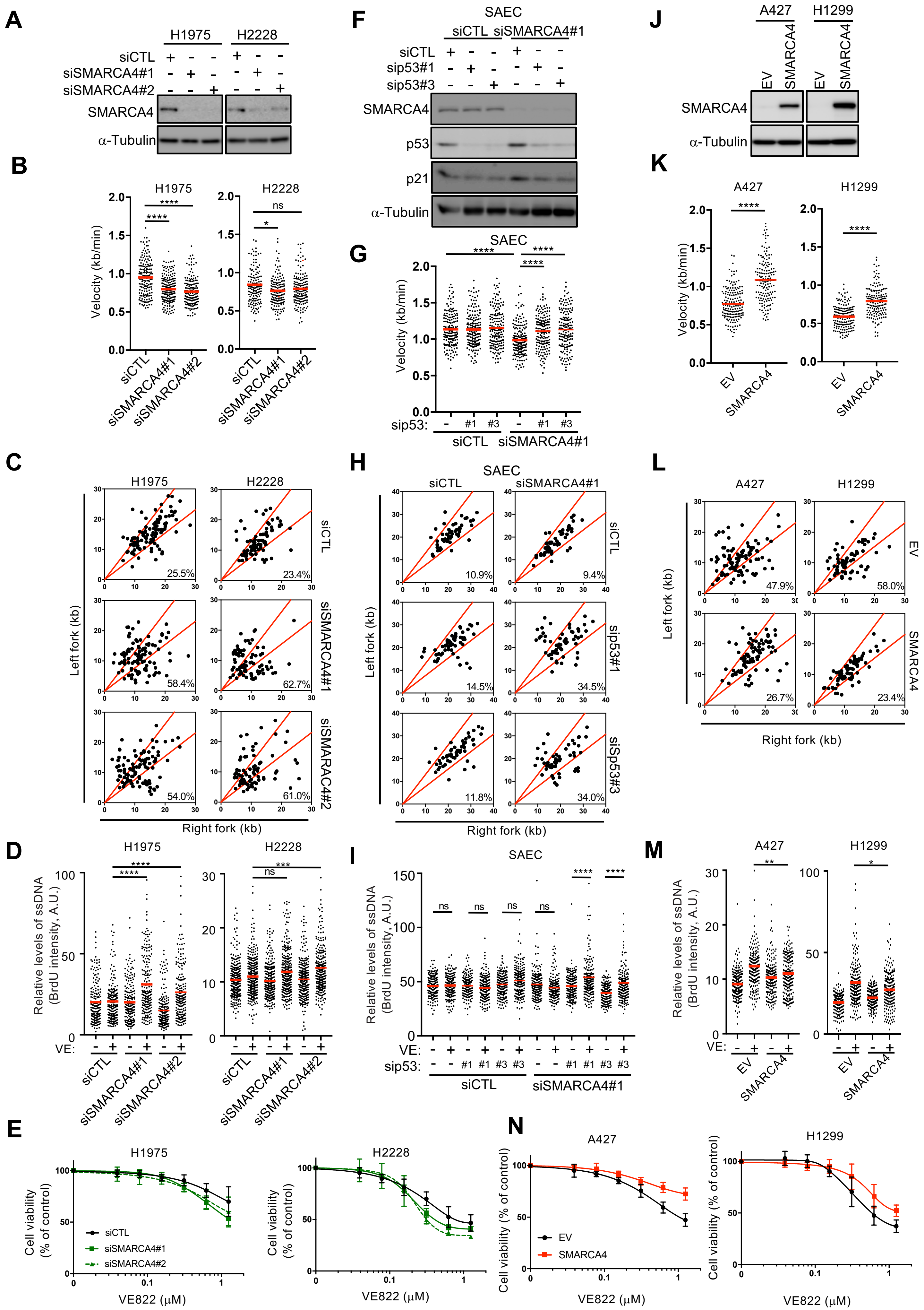

Kurashima et al. Supplementary Figure S4

Supplementary Figure S4. SMARCA4 deficiency enhances intrinsic replication stress and ATRi susceptibility. (**A**-**E**) SMARCA4^{WT} (H1975 and H2228) cells were transfected with control siRNA (siCTL) or two independent SMARCA4 siRNAs. Forty-eight hours after transfection, WCLs were prepared and the SMARCA4 levels were analyzed via western blotting (A). The velocities (B) and asymmetry (C) of individual forks were analyzed via DNA fiber assays. Representative results of two independent reproducible experiments are shown. (D) The exposed ssDNA intensities were analyzed. Quantification of the ssDNA intensities of 200 S-phase cells is shown. Red bars represent the mean intensities. Representative results of two independent reproducible experiments are shown. (E) Forty-eight hours after transfection with siCTL and siSMARCA4, the cells were re-seeded, incubated for 24 h, and treated with increasing ATRi doses, and cell viability was measured via the PrestBlue assay. The values represent the mean $\pm$ S.D. of three independent experiments. (**F**-**I**) SAECs were transfected with the indicated siRNAs. Forty-eight hours after transfection, WCLs were prepared and the SMARCA4, p53 and p21 levels were analyzed via western blotting(F). The velocities (G) and asymmetry (H) of individual forks were analyzed via DNA fiber assays. Representative results of two independent reproducible experiments are shown. (I) Quantification of the ssDNA intensities of 200 S-phase cells is shown. Red bars represent the mean intensities. Representative results of two independent reproducible experiments are shown. **(J**-**N**) SMARCA4^{MUT} cells (A427 and H1299) were lentiviraly transduced with SMARCA4 or empty vector (EV). WCLs were prepared, and the SMARCA4 levels were analyzed via western blotting (J). The fork velocities (K) and asymmetry (L) of individual DNA fibers were analyzed. Representative results of two independent reproducible experiments are shown.(M) Quantification of the ssDNA intensities of 200 S-phase is shown. Red bars represent the mean intensities. Representative results of two independent reproducible experiments are shown. (N) The cells were treated with increasing ATRi doses, and cell viability was measured via the PrestBlue assay. The values represent the mean $\pm$ S. D. of three independent experiments.

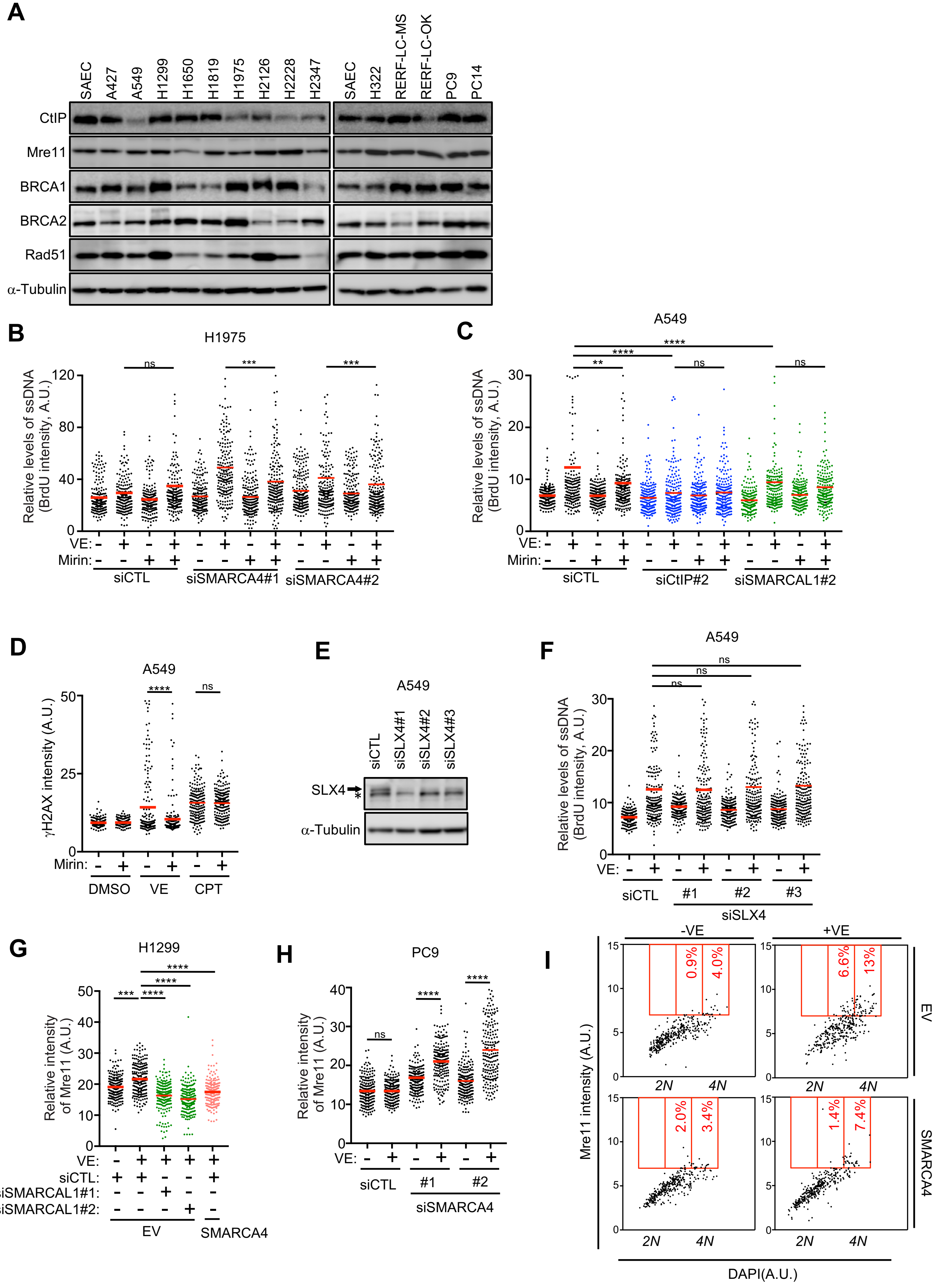

Kurashima et al. Supplementary Figure S5

Supplementary Figure S5. ATRi-induced ssDNA formation depends on replication fork reversal and Mre11 activity.

(**A**) WCLs of SAECs and LADC cells were prepared, and the CtIP, Mre11, BRCA1, BRCA2, and Rad51 levels were analyzed via western blotting. α-Tubulin was used as the loading control. (**B**) Exposed ssDNA was evaluated in $SMARCA4^{WT}$ H1975 cells transfected with siCTL or two independent SMARCA4 siRNAs for 48 h and pretreated or not treated with Mre11i (1 h, 50 μM) followed by ATRi treatment (2 h, 1μM). Quantification of the exposed ssDNA intensities of 200 S-phase cells is indicated. Red bars represent the mean intensities. Representative results of two independent reproducible experiments are shown. (**C**) Quantification of the exposed ssDNA intensities of 200 S-phase A549 cells transfected with the indicated siRNAs and treated with ATRi and/or Mre11i is shown. Red bars represent the mean intensities. Representative results of two independent reproducible experiments are shown. (**D**) A549 cells were pretreated or not pretreated with Mre11i for 1 h followed by treatment with ATRi (1 μM) or CPT (1 μM). Quantification of the γH2AX intensities of 200 S-phase cells is shown. Red bars represent the mean intensities. Representative results of two independent reproducible experiments are shown. (**E**) A549 cells were transfected with siCTL or three independent siSLX4s for 48 h, and the SLX4 (arrow) levels were analyzed via western blotting. *Non-specific band. (**F**) A549 cells were transfected with the indicated siRNAs and treated with ATRi for 2 h. Quantification of the exposed ssDNA intensities of 200 S-phase cells is shown. Red bars represent the mean intensities. Representative results of two independent reproducible experiments are shown. (**G**) H1299 cells transduced with EV or SMARCA4 were transfected with the indicated siRNAs and treated with 1 μM ATRi for 2 h. Quantification of the total Mre11 intensities of 200 S-phase cells is shown. Red bars represent the mean intensities. Representative results of two independent reproducible experiments are shown. (**H**) PC9 cells transfected with the indicated siRNAs. After 48 h later, cells were treated with 1 μM ATRi for 2 h. Quantification of the total Mre11 intensities of 200 S-phase cells is shown. Red bars represent the mean intensities. Representative results of two independent reproducible experiments are shown. (**I**) Scatter plots of total intensity of Mre11 versus DNA content (DAPI intensity) in Figure 5F. Percentages of cells positive for Mre11 in early-, middle- and late-S phases (Red regions) are indicated. Representative results of two independent reproducible experiments are shown.

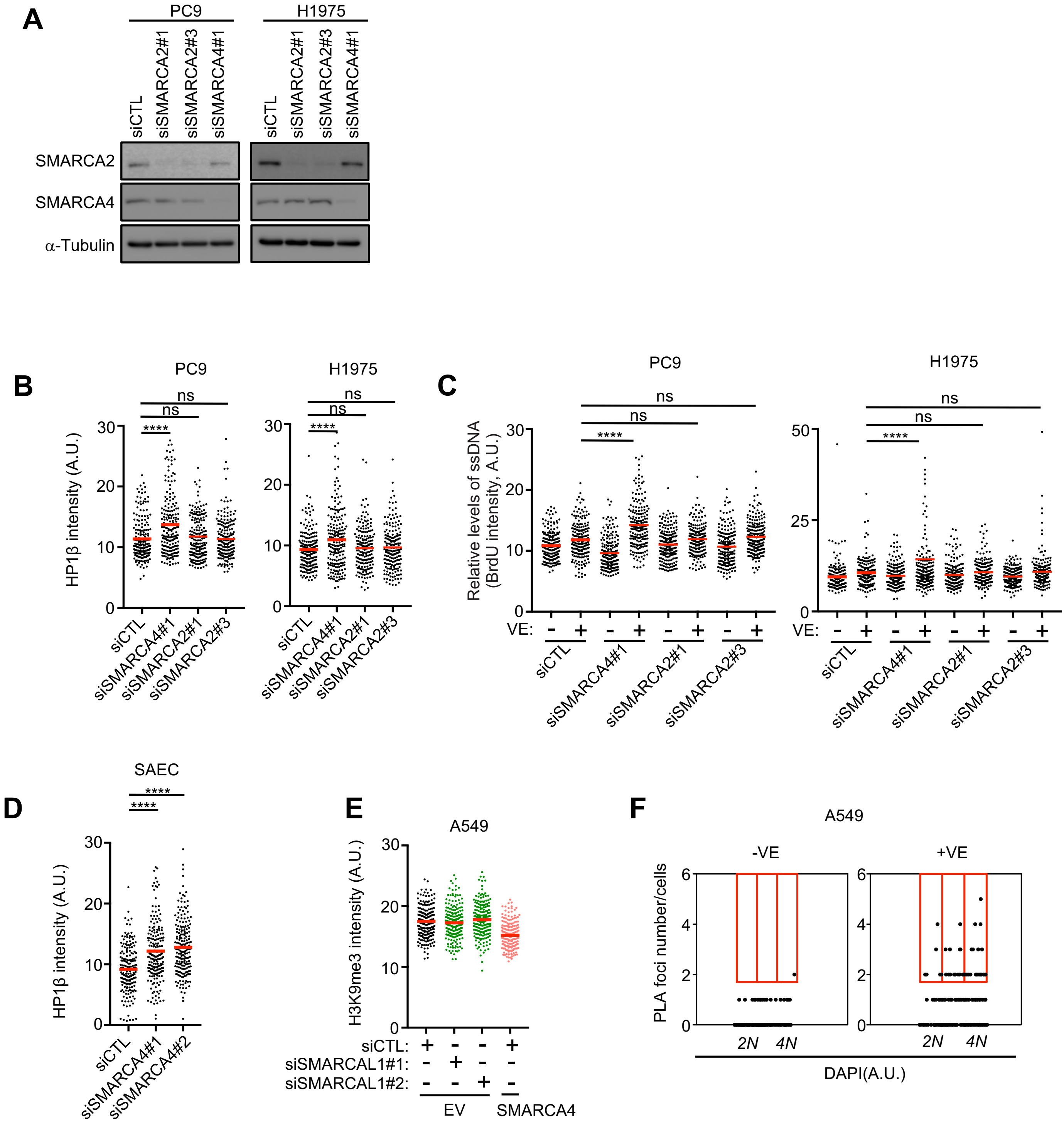

Kurashima et al. Supplementary Figure S6

Supplementary Figure S6. Depletion of SMARCA2 dose not increase HP1β and ATRi-induced ssDNA.

(**A**) SMARCA4^{WT} (PC9 and H1975) cells were transfected with siCTL, SMARCA4 siRNA #1 or two independent SMARCA2 siRNAs. Forty-eight hours after transfection, WCLs were prepared and the SMARCA2 and SMARCA4 levels were analyzed via western blotting. (**B**) PC9 and H1975 cells were transfected with siCTL, siSMARCA2 or siSAMRCA4 for 48 h and immunostained with anti-HP1β antibody. Quantification of the HP1β intensities of 200 cells is shown. Red bars represent the mean intensities. Representative results of two independent reproducible experiments are shown. (**C**) In PC9 and H1975 cells transfected with siCTL, siSMARCA2 or siSAMRCA4, the exposed ssDNA intensities were analyzed. Quantification of the ssDNA intensities of 200 S-phase cells is shown. Red bars represent the mean intensities. Representative results of two independent reproducible experiments are shown. (D) SAECs were transfected with siCTL or two independent siSAMRCA4s for 48 h and immunostained with anti-HP1β antibody. Quantification of the HP1β intensities of 200 cells is shown. Red bars represent the mean intensities. Representative results of two independent reproducible experiments are shown. (**E**) A549 cells transduced with EV or SMARCA4 transfected with the indicated siRNAs. After 48 h later, cells were immunostained with H3K9me3 antibody. Quantification of the total H3K9me3 intensities of 200 cells is shown. Red bars represent the mean intensities. Representative results of two independent reproducible experiments are shown. (**F**) A549 cells were incubated with 10 μM EdU for 15 min and treated with 1 μM ATRi for 2 h. After EdU staining, PLA assays using anti-HP1α and anti-SMARCAL1 antibodies were performed. The nuclei were counterstained with DAPI. Scatter plots of PLA foci number versus DNA content (DAPI intensity) in Figure 6G were shown. Red regions represent the cells positive for PLA signal (2 or more PLA foci/cells) in early-, middle- and late-S phases. Representative results of two independent reproducible experiments are shown.

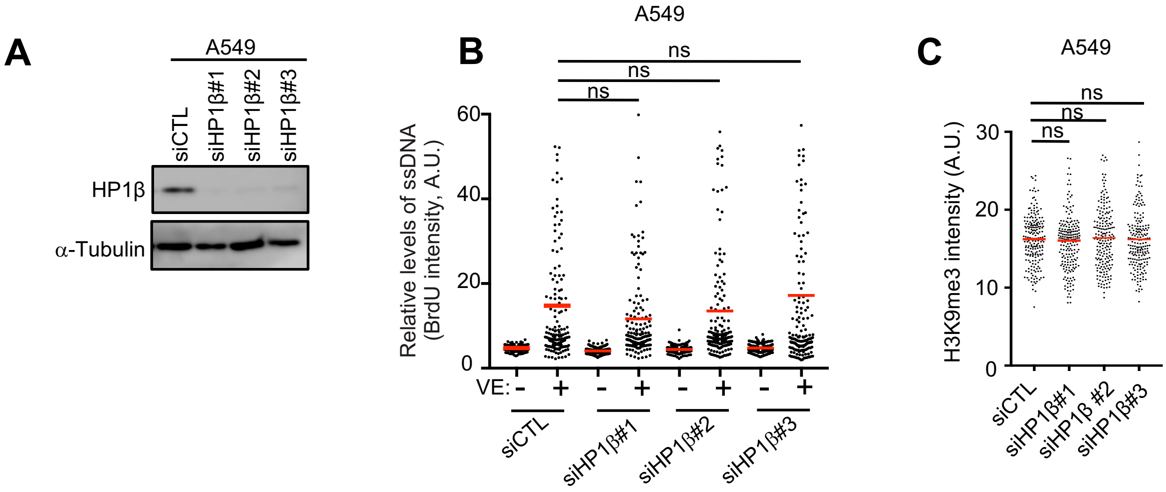

Supplementary Figure S7. Depletion of the HP1β does not reduce ATRi-induced ssDNA in SMARCA4^{MUT} Cells.
(**A**) A549 cells were transfected with siCTL or three independent HP1β siRNAs. Forty-eight hours after transfection, WCLs were prepared and the HP1β levels were sanalyzed via western blotting. (**B**) In A549 cells transfected with siCTL or siHP1β, the exposed ssDNA intensities were analyzed. Quantification of the ssDNA intensities of 200 S-phase cells is shown. Red bars represent the mean intensities. Representative results of two independent reproducible experiments are shown. (**C**) A549 cells were transfected with siCTL or three independent siHP1βs for 48 h and immunostained with antiH3K9me3 antibody . Quantification of H3K9me3 intensities of 200 cells is shown. Red bars represent the mean intensities. Representative results of two independent reproducible experiments are shown.

Supplementary Table S1. List of the cell lines used in this study.

Cell line	Ethnicity	Distributor*	No.
SAEC	Caucasian‡	Lonza	CC-2547
PC-9	Japanese	RIKEN BRC	RCB4455
PC-14	Japanese	IBL	Upon request to us.
RERF-LC-MS	Japanese	JCRB	JCRB0081
RERF-LC-OK	Japanese	JCRB	JCRB0811
A549	Caucasian†	ATCC	CCL-185
A427	Caucasian†	ATCC	HTB-53
H322	Caucasian†	ATCC	CRL-5806
H2228	Unknown†	ATCC	CRL-5935
H1299	Caucasian†	ATCC	CRL-5803
H1650	Caucasian†	ATCC	CRL-5883
H1819	Caucasian†	ATCC	CRL-5897
H1975	Unknown†	ATCC	CRL-5908
H2126	Caucasian†	ATCC	CCL-256
H2347	Caucasian†	ATCC	CRL-5942

*IBL: Immuno-Biological Laboratories; JCRB: Japanese Collection of Research Bioresources; RIKEN BRC: RIKEN Bio Resource Center; ATCC: American Type Culture Collection.

† Refer to the ATCC webpage (http://www.atcc.org/).

‡ Refer to the Lonza webpage (https://www.lonza.com).

Supplementary Table S2. Gene mutations and protein expressions in SAEC and LADC cells.

		SMARCA4		ARID1A		ATM		p53		Putative Driver mutation	
Type	Cell line	Mutation	Protein	Mutation	Protein	Mutation	Protein	Mutation	Protein	Mutation	Gene
Normal	SAEC	WT	Present	WT	Present	WT	Present	WT	Present		-
Cancer	A427	Del at GB	Absent	WT	Present	WT	Present	WT	Present	G12D	K-ras
Cancer	A549	L728fs	Absent	WT	Present	WT	Present	WT	Present	G12S	K-ras
Cancer	H1299	K578fs	Absent	WT	Present	WT	Present	Del at GB	Absent	Q61R	N-ras
Cancer	H1650	N1164Y	Present	WT	Present	WT	Present	SNV at SS	Absent	E746_A750del	EGFR
Cancer	H1819	L1085fs	Absent	WT	Present	WT	Present	SNV at SS	Absent		ERBB-amp
Cancer	H1975	WT	Present	WT	Present	WT	Present	R273H	Present	L858R, T790M	EGFR
Cancer	H2126	W764R	Present	WT	Present	D1853N	Present	E62*	Absent		-
Cancer	H2228	WT	Present	WT	Present	WT	Present	Q331*	Present	EML4-ALK	ALK-fusion
Cancer	H2347	WT	Present	WT	Present	WT	Present	Aberrant splicing	Absent	Q61R	N-ras
Cancer	H322	Del at TSS	Absent	WT	Present	WT	Present	R248L	Absent		-
Cancer	RERF-LC-MS	Del at GB	Present	WT	Present	WT	Present	F134fs	Present		-
Cancer	RERF-LC-OK	WT	Present	WT	Present	WT	Present	F113C	Present		-
Cancer	PC9	WT	Present	WT	Present	WT	Present	R248Q	Present	E746_A750del	EGFR
Cancer	PC14	Gain at GB	Present	WT	Present	WT	Present	R248W	Present	Q61K	N-ras

WT: Wild type
Del: Deletion
fs; Frame Shift
GB: Gene body
TSS: Transcription stating site
SNV; Single nucleotide variation
SS: Splice site
*: Stop codon
